# The long noncoding RNA SNHG1 regulates colorectal cancer cell growth through interactions with EZH2 and miR-154-5p

**DOI:** 10.1186/s12943-018-0894-x

**Published:** 2018-09-28

**Authors:** Mu Xu, Xiaoxiang Chen, Kang Lin, Kaixuan Zeng, Xiangxiang Liu, Bei Pan, Xueni Xu, Tao Xu, Xiuxiu Hu, Li Sun, Bangshun He, Yuqin Pan, Huiling Sun, Shukui Wang

**Affiliations:** 1 General Clinical Research Center, Nanjing First Hospital, Nanjing Medical University, No. 68, Changle Road, Nanjing, 210006 China; 2 School of Medicine, Southeast University, Nanjing, 210009 China; 3 Department of Laboratory Medicine, Nanjing First Hospital, Nanjing Medical University, Nanjing, 210006 China; 4 Department of Laboratory Medicine, The Second Affiliated Hospital of Nanjing Medical University, Nanjing, 210011 China

**Keywords:** SNHG1, PRC2, miR-154-5p, Colorectal cancer

## Abstract

**Background:**

Mounting evidence demonstrates that long noncoding RNAs (lncRNAs) have critical roles during the initiation and progression of cancers. In this study, we report that the small nucleolar RNA host gene 1 (SNHG1) is involved in colorectal cancer progression.

**Methods:**

We analyzed RNA sequencing data to explore abnormally expressed lncRNAs in colorectal cancer. The effects of SNHG1 on colorectal cancer were investigated through in vitro and in vivo assays (i.e., CCK-8 assay, colony formation assay, flow cytometry assay, EdU assay, xenograft model, immunohistochemistry, and western blot). The mechanism of SNHG1 action was explored through bioinformatics, RNA fluorescence in situ hybridization, luciferase reporter assay, RNA pull-down assay, chromatin immunoprecipitation assay and RNA immunoprecipitation assay.

**Results:**

Our analysis revealed that SNHG1 was upregulated in human colorectal cancer tissues, and high SNHG1 expression was associated with reduced patient survival. We also found that high SNHG1 expression was partly induced by SP1. Moreover, SNHG1 knockdown significantly repressed colorectal cancer cells growth both in vitro and in vivo*.* Mechanistic investigations demonstrated that SNHG1 could directly interact with Polycomb Repressive Complex 2 (PRC2) and modulate the histone methylation of promoter of Kruppel like factor 2 (KLF2) and Cyclin dependent kinase inhibitor 2B (CDKN2B) in the nucleus. In the cytoplasm, SNHG1 acted as a sponge for miR-154-5p, reducing its ability to repress Cyclin D2 (CCND2) expression.

**Conclusions:**

Taken together, the results of our studies illuminate how SNHG1 formed a regulatory network to confer an oncogenic function in colorectal cancer and suggest that SNHG1 may serve as a potential target for colorectal cancer diagnosis and treatment.

**Electronic supplementary material:**

The online version of this article (10.1186/s12943-018-0894-x) contains supplementary material, which is available to authorized users.

## Background

Colorectal cancer (CRC) is the third most commonly-diagnosed cancer type and the fourth leading cause of cancer mortality worldwide [1]. Although, recent technological advances in early detection and intervention have partially improved the overall survival of colorectal cancer, prognosis remains poor for those with advanced stage colorectal cancer given the high frequency of recurrence and metastasis [2, 3]. Therefore, deep molecular mechanisms of colorectal cancer tumorigenesis and progression should be investigated to improve the early diagnosis and treatment.

Advances in whole-genome sequencing technology revealed that greater than 90 % of the human genome is actively transcribed. However, only 2 % of the transcripts encode proteins, and most transcripts are non-coding RNAs (ncRNAs) [4]. Among those non-coding transcripts, lncRNAs have attracted increasing attention. LncRNA is a type of noncoding RNAs that is greater than 200 nucleotides in length. Many studies revealed that lncRNAs could be vital regulators in numerous biological processes, such as cell cycle control, cell differentiation, X chromosome inactivation, mRNA alternative splicing, RNA decay and translation [5, 6]. Importantly, the lncRNA dysregulation has been observed in various diseases including cancer, and lncRNAs are widely reported to participate in cancer cell growth, metastasis and drug resistance [7, 8].

Recently, some lncRNAs have been reported to regulate gene expression through binding to PRC2 in some biological processes [9, 10]. PRC2 is one of the two Polycomb group (PcG) proteins complexes that induce gene silencing via histone modification. As a multisubunit complex, PRC2 is composed of EZH2, EED and SUZ12. Among them, EZH2 is a histone methyltransferase that can catalyze histone H3 lysine 27 trimethylation (H3K27me3) and epigenetically silence target genes [11]. Accumulating evidence reveals that PRC2 plays a vital role in cancer initiation and progression. EZH2 is overexpressed in many cancer types and is proved to act as an oncogene [12, 13].

In this study, we aimed to identify and characterize lncRNAs functionally impacting on colorectal cancer. By analyzing datasets downloaded from The Cancer Genome Atlas (TCGA) and Gene Expression Omnibus (GEO), we found lncRNA SNHG1 was significantly overexpressed in colorectal cancer. In addition, we proved SNHG1 exhibited an oncogenic role in colorectal tumorigenesis, its expression could affect colorectal cancer cells growth in vivo and in vitro. Mechanistic investigations revealed that SNHG1 could exhibit different regulatory mechanisms in the nucleus and cytoplasm, and it could promote the development of colorectal cancer by regulating CCND2, KLF2 and CDKN2B expression.

## Methods

### Cell lines

The human colorectal cancer cell lines (HCT-116, HCT-8, SW-480, SW-620, DLD-1 and HT-29) and the human normal colorectal epithelial cell (FHC) were obtained from American Type Culture Collection. The SW-480, SW-620, DLD-1, HT-29 and FHC cells were cultured in Dulbecco’s modified Eagle’s medium (DMEM) with 10% fetal bovine serum. HCT-116 and HCT-8 cells were cultured in RPMI-1640 with 10% fetal bovine serum. Cells were maintained in a humidified atmosphere of 5% CO_2_ at 37 °C.

### Plasmids construction and cell transfection

Full-length complementary cDNAs of human SP1 and SNHG1 were synthesized and cloned into the expression vector pcDNA3.1 (Invitrogen, China). The small hairpin RNA (shRNA) of SNHG1 was synthesized and cloned into the pLVX-shRNA1 vector (GeneCreat, China). SP1, CCND2, EZH2, KLF2 and CDKN2B siRNAs were synthesized by GenePharma (China). SNHG1 siRNAs were designed and synthesized by Ambion (USA). MicroRNA mimics and inhibitors were purchased from RiboBio (China). The plasmid vectors and siRNAs were transfected into colorectal cancer cells using Lipofectamine 2000 (Invitrogen, USA) according to the manufacturer’s instructions. All siRNA and shRNA sequences are listed in Additional file 1: Table S1.

### Luciferase reporter assay

For the SNHG1 promoter luciferase reporter assay, the core promoter of the SNHG1 gene (− 201 to + 150, relative to the transcription start site of the SNHG1 gene, contained both E1 and E2), promoter region only contained E1 and promoter region only contained E2 were synthesized and cloned into the pGL3-basic firefly luciferase reporter (GeneCreat, China). The pRL-TK vector was employed as a control. For microRNA target gene luciferase reporter assays, target sequences containing predicted microRNA binding sites were respectively synthesized and inserted into the pmirGLO luciferase vector (GeneCreat, China). Luciferase activity was measured with the Dual Luciferase Assay system (Promega, USA). Renilla luciferase activity was normalized to firefly luciferase activity.

### Chromatin immunoprecipitation assay

ChIP assays were performed using the ChIP Assay Kit (Beyotime, China) according to the manual with slight modifications. Briefly, HCT-116 and HCT-8 cells were cross-linked with 1% formaldehyde solution for 10 min at room temperature and quenched with 125 mM glycine. DNA fragments ranging from 200 to 500 bp were yielded via sonication. Then the lysates were immunoprecipitated with anti-SP1, anti-EZH2, anti-H3K27me3 or normal rabbit IgG antibody. Immunoprecipitated DNAs were analyzed by qRT-PCR. Human α Satellite Repeat Primers and Human DHFR Intron 1 Primers were purchased from Cell Signaling Technology (USA). ChIP primers are listed in Additional file 2: Table S2. Antibodies used in ChIP are listed in Additional file 3: Table S3.

### RNA fluorescence in situ hybridization (FISH)

FISH assays were performed using Fluorescent In Situ Hybridization Kit (RiboBio, China) according to the protocol. Cy3-labeled SNHG1 antisense probe 1 and cy3-labeled SNHG1 sense probe were designed and synthesized by RiboBio (China). Cy3-labeled SNHG1 antisense probe 2 was designed and synthesized by GenePharma (China). Cells were first fixed in 4% formaldehyde for 15 min. Then the cells were permeabilized in PBS containing 0.5% Triton X-100 at 4 °C for 30 min and pre-hybridizated at 37 °C for 30 min in pre-hybridization solution. After that, probes were added in the hybridization solution and incubated with the cells at 37 °C overnight in the dark. The next day, the cells were counterstained with DAPI and imaged.

### RNA pull-down assay

RNA pull-down assays were performed using a Magnetic RNA Protein Pull-Down Kit (Pierce, USA) according to the manual. Biotinylated SNHG1 RNA were synthesized by RiboBio (China). For each assay, 50 pmol biotinylated RNA were incubated with 50 μl prewashed streptavidin-agarose beads for one hour at 4 °C. Then, RNA-bound beads were incubated with lysates from CRC cells cytosolic/nuclear extracts and eluted proteins were detected by western blot. 3′ untranslated-region of androgen receptor (AR) RNA which contained UC-rich regions for HuR was employed as a positive control. Poly(A)_25_ RNA did not contain HuR binding sites was employed as a negative control.

### RNA immunoprecipitation assay

The EZ Magna RNA immunoprecipitation Kit (Millipore, USA) was used following the guidelines. Briefly, HCT-116 and HCT-8 cells were lysed in RIP lysis buffer. Magnetic beads were pre-incubated with antibodies for 30 min at room temperature and the cell lysates was immunoprecipitated with beads for 6 h at 4 °C. Then, RNA was purified and detected by qRT-PCR. Antibodies information are listed in Additional file 3: Table S3.

### Tumor xenografts in animals

Five-week-old male BALB/c nude mice were maintained under specific pathogen-free conditions and manipulated according to protocols approved by the Animal Care Committee of Nanjing Medical College. Stably transfected HCT-116 cells (5 × 10^6^/0.2 ml PBS) were implanted into two sides of the same nude mouse in the armpit. Xenografts were examined every 3 days with digital calipers and tumor volumes were calculated using the following equation: volume = 1/2 (length × width^2^). Sixteen days later, the mice were sacrificed, and tumors volumes were measured. The samples were embedded in paraffin for hematoxylin and eosin (HE) staining and immunohistochemistry staining.

### Gene set enrichment analysis

Gene set enrichment analysis (GSEA) was used to explore pathways and gene sets associated with SNHG1 in colorectal cancer. Gene expression profiles of 481 colorectal cancer samples were downloaded from TCGA dataset. According to the SNHG1 expression level order, the top 25 % and the bottom 25 % of samples were grouped as high SNHG1 group and low SNHG1 group respectively. GSEA v3.0 was used to determine whether the members of the gene sets from the MSigDB database are randomly distributed at the top or bottom of the ranking [14]. If most members of a gene set were positively related to the SNHG1, the set was termed associated with SNHG1.

### Statistical analysis

All statistical analyses were performed using SPSS 18.0 (SPSS, USA) and GraphPad Prism 6 (GraphPad, USA) software. A chi-square test was used to analyze the different distribution of clinical variables. Differences in gene expression levels were analyzed using the student’s t-test. Univariate and Multivariate Cox proportional hazards regression models were used to analyze potential factors associated with prognosis. Overall survival was estimated with the Kaplan–Meier method and log-rank test was employed to evaluate difference. For in vitro and in vivo experiments, the t-test or analysis of variance (ANOVA) was used to evaluate the difference between different groups. All *P*-values were two sided, and *P* < 0.05 was statistically significant. All data are presented as the mean ± standard deviation (SD) from at least three independent replicates.

A complete description of the methods, including tissue samples and clinical data collection, RNA isolation and quantitative reverse transcription polymerase chain reaction, protein extraction and western blot, flow cytometry, cell growth and colony formation assays, 5-Ethynyl-20-deoxyuridine (EdU) incorporation assay, subcellular fractionation location and immunohistochemistry are available in Additional file 4: Supplementary materials and methods.

## Results

### SNHG1 is up-regulated in human colorectal cancer

**Fig. 1:**
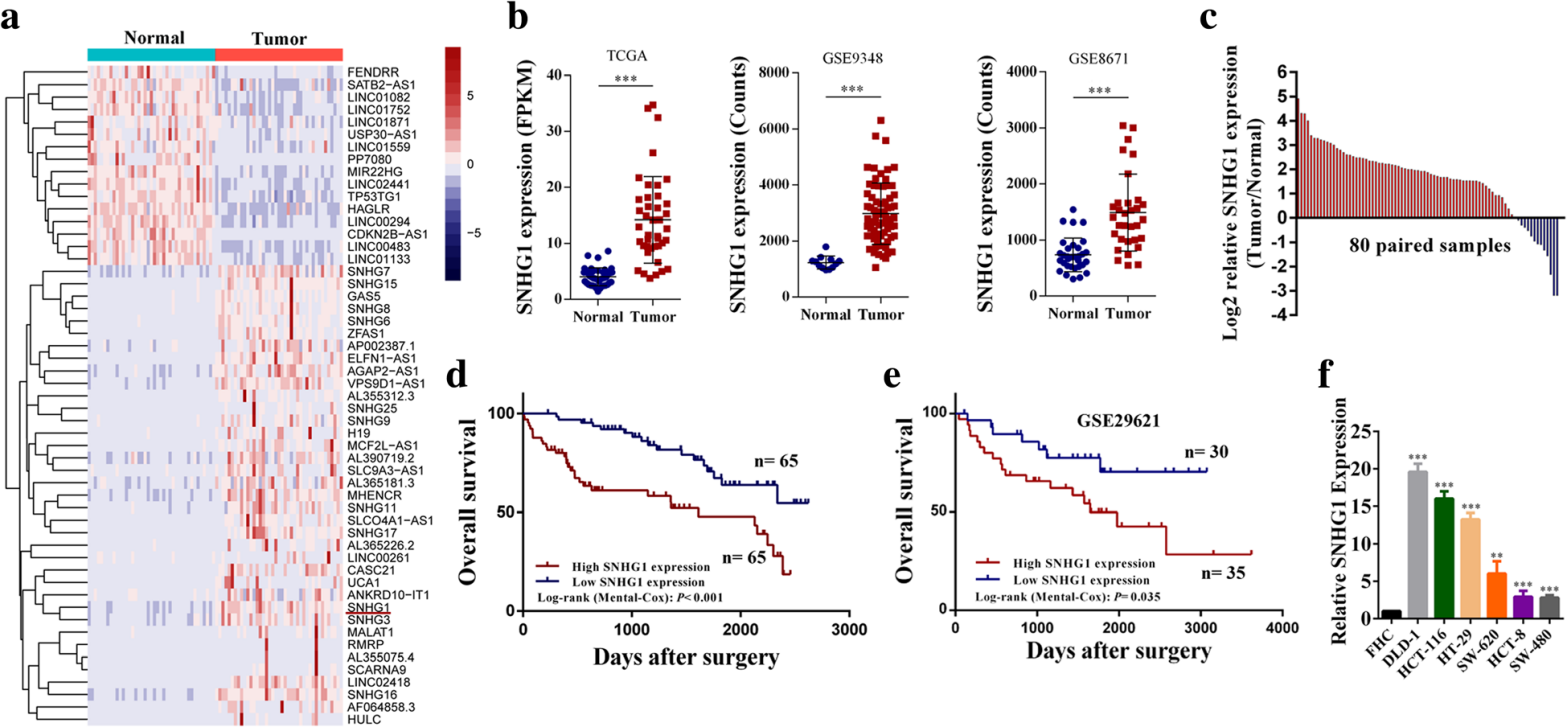
SNHG1 expression is up-regulated in colorectal cancer and is correlated with prognosis. **a** Hierarchical cluster heat map of differentially expressed lncRNAs in colorectal cancer and corresponding normal tissues generated from RNA sequencing data from the TCGA database. Red in the heat map denotes upregulation; blue denotes downregulation. The red arrow indicates SNHG1. **b** Expression of SNHG1 in the TCGA, GSE9348 and GSE8671 cohorts. **c** qRT-PCR analysis of SNHG1 expression in 80 pairs of colorectal cancer and corresponding normal tissues. **d** Kaplan-Meier survival analysis of CRC patients’ overall survival based on SNHG1 expression in our cohort (*n* = 130, *P* < 0.001). **e** Kaplan-Meier survival analysis of CRC patients’ overall survival based on SNHG1 expression in GSE29621 (*n* = 65, *P* = 0.035). **f** SNHG1 expression in colorectal cancer cell lines (DLD-1, HCT-116, HT-29, SW-620, HCT-8 and SW-480) compared with normal colorectal epithelial cells FHC detected by qRT-PCR. **P < 0.01 and ***P < 0.001

In this study, we first analyzed RNA sequencing data of colorectal cancer and para-cancerous tissues downloaded from TCGA [15]. As expected, many lncRNAs were identified to be aberrantly expressed in colorectal cancer (Fig. 1a). Among these differentially expressed lncRNAs, we focused on SNHG1 given its abundance is relatively high. We then analyzed two independent microarray datasets downloaded from GEO [16, 17], SNHG1 is also significantly upregulated in colorectal cancer (Fig. 1b). Similar results were also observed in 80 paired colorectal cancer tissues and adjacent tissues analyzed by qRT-PCR, as shown in Fig. 1c, SNHG1 was significantly up-regulated in 83.8% (67 of 80) of colorectal cancer tissues. To explore the correlation between SNHG1 expression and clinic-pathological features, we divided enrolled patients into two groups based on SNHG1 expression value. Chi-square test was performed to analyze clinical characters between the two groups. As shown in Table 1, SNHG1 level was correlated with tumor invasion depth (*P* = 0.035), distant metastasis *(P =* 0.024*)* and TNM stage (*P* = 0.006). Besides, the SNHG1 expression in different tumor stages of colorectal cancer in TCGA cohort was shown in (Additional file 5: Figure S1a). We also investigated the relationship between SNHG1 expression and colorectal cancer prognosis. Survival analysis using the Kaplan–Meier method revealed that a higher SNHG1 level was associated with reduced overall survival in CRC patient (*P <* 0.001) (Fig. 1e). The same result was identified in an independent GEO dataset [18] (*P =* 0.035) and in TCGA cohort (*P* = 0.099) (Fig. 1d and Additional file 5: Figure S1b). To further confirm the prognostic role of SNHG1 expression in colorectal cancer, the univariate and multivariate survival analysis were performed. As shown in Table 2, univariate analysis identified three prognostic factors: SNHG1 expression (*P <* 0.001), TNM stage (*P <* 0.001) and distant metastasis (*P <* 0.001). In addition, multivariate analysis of the three prognosis factors revealed that SNHG1 expression was an independent prognostic biomarker (Hazard ratio [HR] = 2.82; 95% confidential interval [CI] = 1.53–5.18; *P =* 0.001) for colorectal cancer. Besides, we analyzed SNHG1 expression in human colorectal cancer cell lines. As shown in Fig. 1f, SNHG1 expression was upregulated in all six colorectal cancer cell lines (HCT-29, DLD-1, SW-620, HCT-8, HCT-116 and SW-480) compared with the human colorectal epithelial cell FHC. In addition, per cell copy numbers of SNHG1 in these cell lines were detected (Additional file 5: Figure S1c). Moreover, through analyses of RNA sequencing data in TCGA, we found SNHG1 was upregulated in most cancers (Additional file 5: Figure S1d). These results indicate that SNHG1 upregulation may play a critical role in the development and progression of colorectal cancer.

**Table 1:** The clinic-pathological factors of 130 CRC patients for survival analysis

Characteristics	Number of cases	SNHG1 expression		*P* value^a^
		Low (*n* = 65)	High (*n* = 65)	
Age(year)				
< 60	57	31	26	0.377
≥ 60	73	34	39	
Gender				
Female	69	35	34	0.860
Male	61	30	31	
Tumor invasion depth				
T1–2	91	51	40	**0.035**
T3–4	39	14	25	
Lymph node metastasis				
N0	103	51	52	0.829
N1 + N2	27	14	13	
Distant metastasis				
M0	106	58	48	**0.024**
M1 + M2	24	7	17	
TNM stage				
I + II	94	54	40	**0.006**
III + III	36	11	25	

^a^Statistical significant results (in bold)

**Table 2:** Univariate and multivariate analysis of clinic pathologic factors for overall survival in 130 CRC patients

Risk factors	Univariate analysis		Multivariate analysis	
	HR (95% CI)	*P* value ^a^	HR (95% CI)	*P* value ^a^
SNHG1 expression (Low vs. High expression)	2.96 (1.64–5.37)	**< 0.001**	2.82 (1.53–5.18)	**0.001**
TNM stage (I/II vs. III/IV)	2.80 (1.59–4.93)	**< 0.001**	1.12 (0.52–2.42)	0.773
Tumor invasion depth (T1*/*T2 vs. T3*/*T4)	1.39 (0.76–2.53)	0.281		
Lymph node metastasis (N0 vs. N1 or above)	1.22 (0.64–2.31)	0.549		
Distant metastasis (M0 vs. M1)	4.69 (2.49–8.83)	**< 0.001**	4.15 (1.76–9.77)	**0.001**
Age (≤60 vs. > 60)	0.99 (0.56–1.72)	0.934		
Gender (Male vs. Female)	0.84 (0.48–1.49)	0.551		

*HR* hazard ratio; *CI* confidential interval; *vs.* versus

^a^Statistical significant results (in bold)

### SP1 activates SNHG1 transcription in colorectal cancer cells

**Fig. 2:**
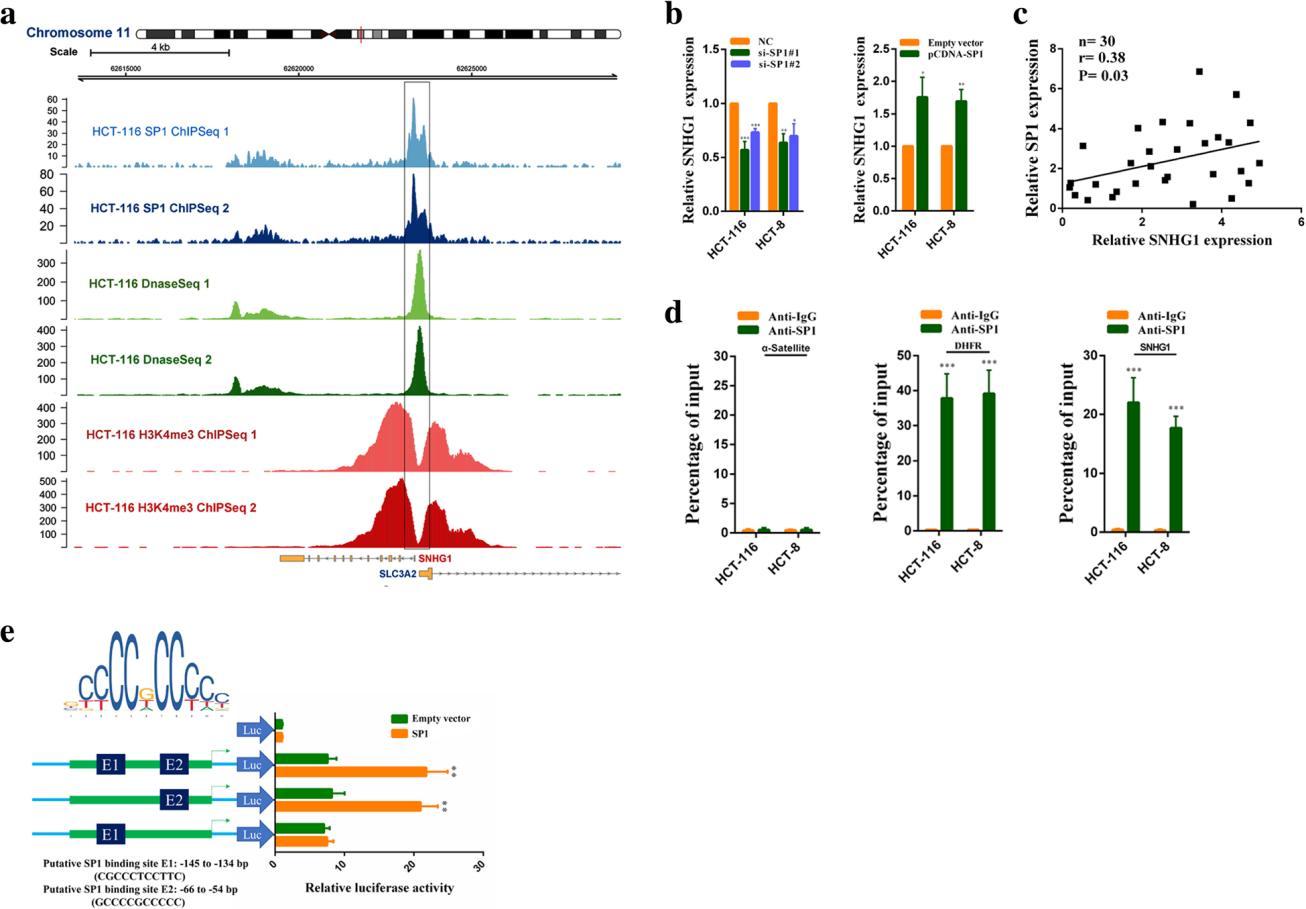
SP1 activates SNHG1 transcription in colorectal cancer cells. **a** Analysis of SP1 ChIP-seq, H3K4me3 ChIP-seq and DnaseI-seq data of HCT-116 cells in the SNHG1 locus. **b** SNHG1 expression was detected by qRT-PCR in HCT-116 and HCT-8 cells transfected with SP siRNAs or the SP1 vector. **c** The correlation between SP1 and SNHG1 expression analyzed in 30 paired colorectal cancer samples (*n* = 30, *r* = 0.38, *P* = 0.03). **d** ChIP assays were performed to detect SP1 occupancy at the SNHG1 promoter region, α-Satellite and DHFR were employed as negative and positive control respectively for SP1 ChIP assays. **e** Dual luciferase reporter assays were used to determine the SP1 binding sites on the SNHG1 promoter region. The upper left corner of the picture was SP1 binding motif provided by the JASPAR CORE database. *P < 0.05, **P < 0.01 and ***P < 0.001

To investigate potential regulators of SNHG1 overexpression in colorectal cancer, we used the JASPAR CORE database to search transcription factor binding sites in SNHG1 promoter [19]. Putative SP1 binding sites (GCCCCGCCCCC, − 66 bp to − 54 bp upstream of transcription start site) got the highest score. We next analyzed ChIP-Seq data of HCT-116 downloaded from the Encyclopedia of DNA Elements (ENCODE) database [20]. As shown in Fig. 2a, SP1 was highly enriched in the SNHG1 promoter region. Immunohistochemistry analysis revealed that SP1 was up-regulated in CRC (Additional file 6: Figure S2a). We then knocked down SP1 in HCT-116 and HCT-8 cells, SNHG1 expression was decreased. Moreover, SP1 overexpression promoted SNHG1 expression (Fig. 2b and Additional file 6: Figure S2b). In addition, we found SNHG1 expression was positively correlated with SP1 expression in colorectal cancer sequencing data from TCGA (Additional file 6: Figure S2c), and the positive correlation was also observed in our samples (Fig. 2c). Furthermore, ChIP assays indicated SP1 bound to the SNHG1 promoter region directly. In SP1 ChIP assays, α-Satellite and DHFR were employed as negative and positive control respectively (Fig. 2d). Besides, luciferase report assays revealed that SP1 bound to the E2 sites (− 66 bp to − 54 bp upstream of transcription start site), but not the E1 sites (− 145 bp to − 134 bp upstream of transcription start site) (Fig. 2e). Overall, above results indicate that SNHG1 overexpression in colorectal cancer is at least partly due to SP1 activation.

### SNHG1 affects growth of colorectal cancer cell

**Fig. 3:**
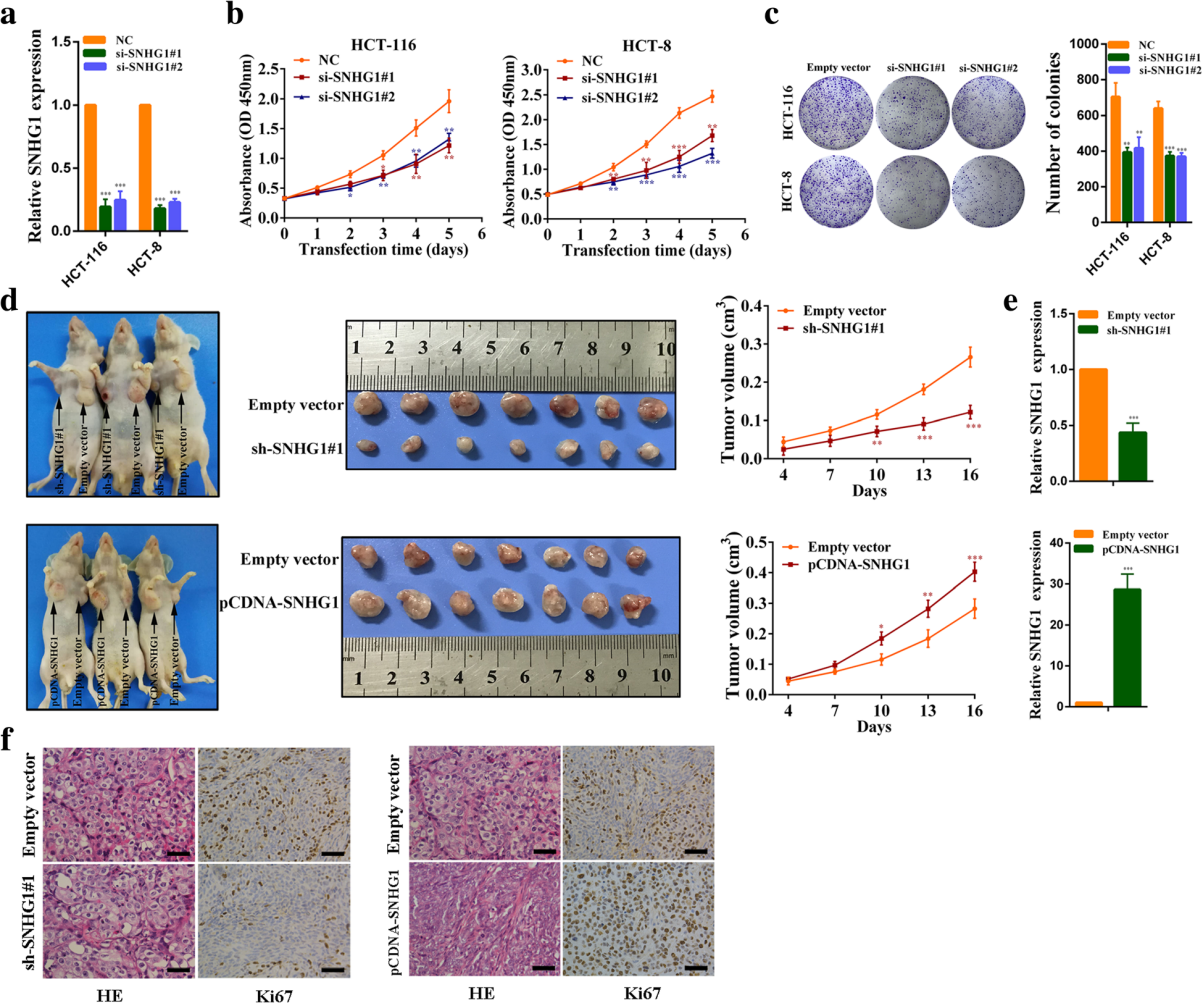
SNHG1 affects colorectal cancer cells growth. **a** SNHG1 expression was detected by qRT-PCR in HCT-116 and HCT-8 cells transfected with two SNHG1 siRNAs. **b** HCT-116 and HCT-8 cells transfected with SNHG1 siRNAs were subjected to the CCK-8 assay after transfection. **c** HCT-116 and HCT-8 cells transfected with SNHG1 siRNAs were seeded onto 6-well plates. The number of colonies was counted on the 14th day after seeding. **d** Representative images of mice bearing tumors from empty vector, sh-SNHG1#1 vector and SNHG1 vector groups, and the tumor volume growth curves after injections in different groups. **e** SNHG1 expression was detected in tumors from different groups of mice using qRT-PCR. **f** Representative images of hematoxylin and eosin (HE) staining and Ki67 immunostaining of tumor samples from different groups. Scale bar = 50 μm. *P < 0.05, **P < 0.01 and ***P < 0.001

We designed two independent small interfering RNAs (siRNAs) to silence SNHG1 expression. As shown in Fig. 3a, SNHG1 expression was strongly reduced when examined 24 h after siRNA transfection in HCT-116 and HCT-8 cells. Next, CCK-8 assays demonstrated that SNHG1 knockdown inhibited cell growth significantly (Fig. 3b). Similarly, clone formation assays showed that clone forming ability of HCT-116 and HCT-8 cells decreased following SNHG1 knockdown (Fig. 3c). We further explored whether SNHG1 could affect colorectal cancer growth in vivo. HCT-116 cells stably transfected with sh-SNHG1#1, pCDNA-SNHG1 or empty vector were injected into male nude mice. Sixteen days after the injection, tumors from the sh-SNHG1#1 group were significantly smaller compared with the control group. Conversely, tumors of the pCDNA-SNHG1 group were significantly larger than those in the control group (Fig. 3d). We performed qPCR analyses to confirm SNHG1 expression in xenografted tumor tissues. As expected, tumors formed from sh-SNHG1#1 cells exhibited reduced SNHG1 expression, whereas tumors that from pCDNA-SNHG1 cells exhibited increased SNHG1 expression (Fig. 3e). Besides, tumor tissues collected from the sh-SNHG1#1 group exhibited lower Ki67-positive rates, whereas the pCDNA-SNHG1 group exhibited higher Ki67-positive rates compared with the control group (Fig. 3f). These findings indicate that SNHG1 can affect colorectal cancer cells growth in vitro and in vivo.

### SNHG1 regulates colorectal cancer cell proliferation and apoptosis

**Fig. 4:**
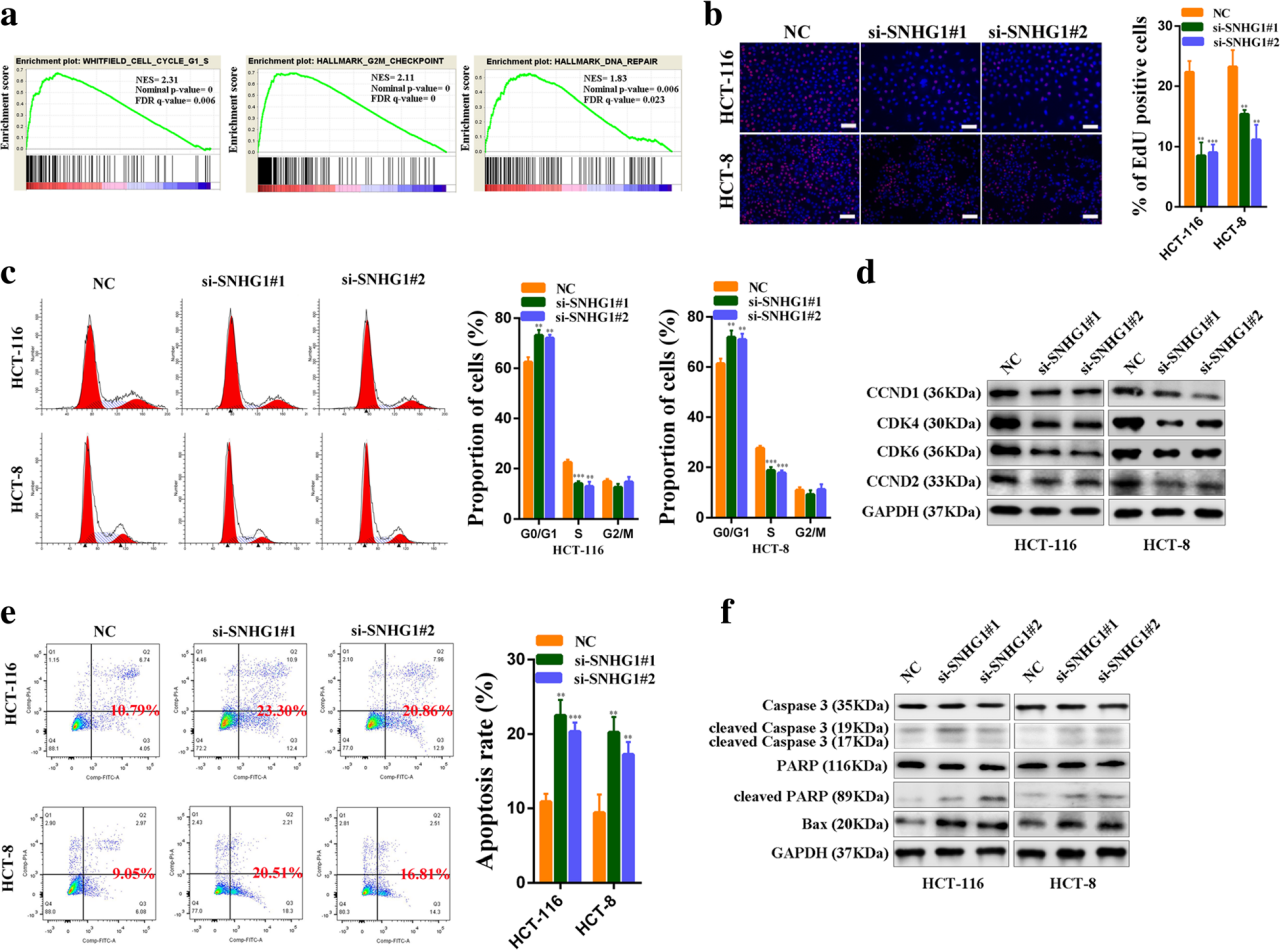
SNHG1 regulates colorectal cancer cell proliferation and apoptosis. **a** Results of gene set enrichment analysis (GSEA) were plotted to visualize the correlation between the expression of SNHG1 and cell cycle and DNA repair gene signatures in TCGA cohort. **b** EdU assays were used to determine the cell proliferation ability of si-SNHG1 transfected cells. **c** Flow cytometric cell cycle distribution assays to detect the proportion of colorectal cancer cell cells in G1, S, and G2/M phases after transfection with SNHG1 siRNAs. **d** The cell cycle related proteins CyclinD1, CDK4, CDK6, and CyclinD2 were detected by western blot following SNHG1 silencing. **e** The effect of SNHG1 knockdown on cell apoptosis was analyzed by flow cytometric cell apoptosis assays. **f** Apoptosis related proteins Caspase-3, cleaved Caspase-3, PARP, cleaved PARP and Bax were detected by western blot after SNHG1 knockdown. Scale bar = 50 μm. **P < 0.01 and ***P < 0.001

Gene Set Enrichment Analysis (GSEA) revealed significant relations between the expression of cell cyclin related genes, DNA repair related genes and SNHG1 in CRC (Fig. 4a). We then employed Ethynyl deoxy Uridine (EdU) dye assays to observe cell proliferation rates. As we hypothesized, SNHG1 knockdown could inhibit colorectal cancer cell proliferation (Fig. 4b). Consistent with the EdU assay results, flow cytometry cell cycle analysis revealed that SNHG1 knockdown decreased the proportion of cells in S phase (Fig. 4c). In addition, we examined cell cycle related proteins by western blot. The results revealed that Cyclin D1, Cyclin D2, CDK4 and CDK6 were all decreased in si-SNHG1-transfected colorectal cancer cells (Fig. 4d). Furthermore, flow cytometry cell apoptosis analysis demonstrated a significantly increasd proportion of apoptotic cells following SNHG1 knockdown (Fig. 4e). Similarly, the expression of apoptosis related proteins, including cleaved Caspase-3, cleaved PARP and Bax, significantly increased when SNHG1 was silenced (Fig. 4f). Taken together, these results indicate that SNHG1 promotes colorectal cancer cell growth by affecting cell cycle progression and apoptosis.

### SNHG1 functions as a sponge for miR-154-5p in the cytoplasm

**Fig. 5:**
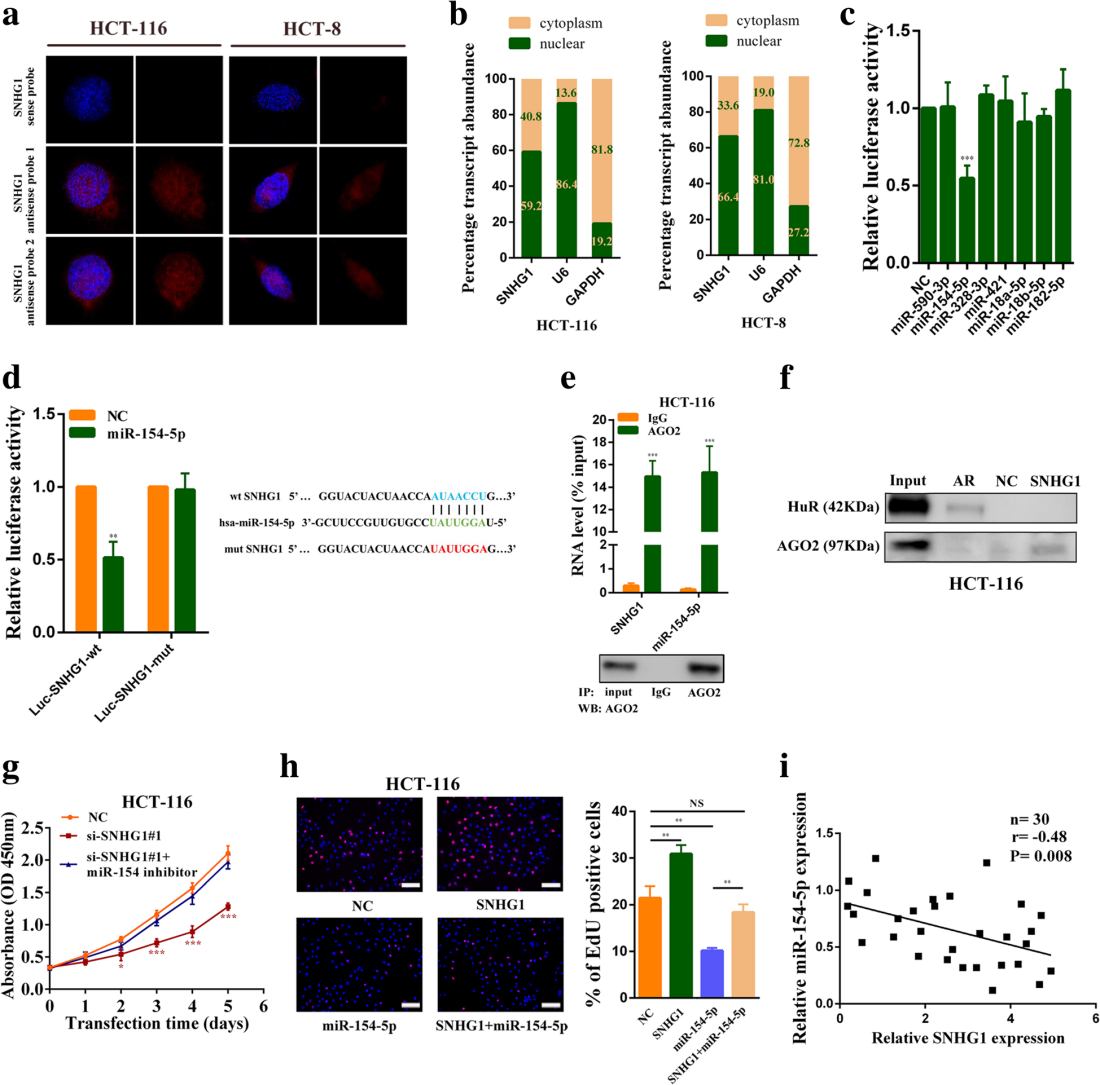
SNHG1 acts as a sponge for miR-154-5p in the cytoplasm. **a** Representative FISH images indicated subcellular location of SNHG1 in HCT-116 and HCT-8 cells (red). Nuclei were stained by DAPI (blue). SNHG1 sense probe was employed as a negative control. **b** Relative SNHG1 expression levels in nuclear and cytosolic fractions of HCT-116 and HCT-8 cells. Nuclear controls: U6; Cytosolic controls: GAPDH. **c** Dual luciferase reporter assays were used to determine miRNAs that directly interacted with SNHG1. Luciferase activity is presented as relative luciferase activity normalized to activity of their respective negative control. **d** Dual luciferase reporter assays were conducted with wild type and mutant type (putative binding sites for miR-154-5p were mutated) luciferase reporter vectors. Right panel, sequence alignment of miR-154-5p and its predicted binding sites (green) in SNHG1. Predicted miR-154-5p target sequence (blue) in SNHG1 (Luc-SNHG1-wt) and position of mutated nucleotides (red) in SNHG1 (Luc-SNHG1-mut). **e** RNA immunoprecipitation with an anti-Ago2 antibody was used to assess endogenous Ago2 binding to RNA in HCT-116 cells, IgG was used as the control. SNHG1 and miR-154-5p levels were determined by qRT–PCR and presented as fold enrichment in Ago2 relative to input. RIP efficiency of Ago2 protein was detected by western blot. **f** RNA pull-down assays were used to examine the interaction of SNHG1 and Ago2 in HCT-116 cells. **g** CCK-8 assays demonstrated that SNHG1 silencing inhibited HCT-116 cell growth. MiR-154-5p down-regulation rescued growth inhibition caused by SNHG1 knockdown. **h** EdU assays revealed that SNHG1 overexpression promotes HCT-116 cell proliferation. Co-transfecting miR-154-5p mimics with the SNHG1 plasmid abolished the increased proliferation rates. **i** The correlation between miR-154-5p and SNHG1 expression analyzed in 30 paired colorectal cancer samples (n = 30, *r* = − 0.48, *P* = 0.008). Scale bar = 50 μm. *P < 0.05, **P < 0.01 and ***P < 0.001

To investigate the potential mechanisms by which SNHG1 contributed to the malignant phenotypes of colorectal cancer cells, we first examined its subcellular localization given that the function of one lncRNA depended on its subcellular distribution [21]. Using fluorescence in situ hybridization (FISH) and subcellular fractionation, we observed that SNHG1 was expressed both in the nucleus and cytoplasm, and a larger proportion of SNHG1 was observed in the nucleus (Fig. 5a and Fig. 5b). Many cytoplasmic lncRNAs have been reported to act as competing endogenous RNAs (ceRNAs) by competitively binding microRNAs. Using StarBase v2.0 software, we found that a set of microRNAs were predicted to have a high probability of binding to SNHG1 [22]. We then used dual luciferase reporter assays to determine which miRNAs could directly interact with SNHG1 in HCT-116 cells. As shown in Fig. 5c, miR-154-5p overexpression significantly reduced the luciferase activity. To further identify whether miR-154-5p could bind to the predicted target sites in SNHG1, we constructed wild type and mutant type (putative binding sites for miR-154-5p were mutated) SNHG1 luciferase reporter vectors. As expected, co-transfection of the wild-type SNHG1 vector (Luc-SNHG1-wt) with miR-154-5p mimics, but not the mutant SNHG1 vector (Luc-SNHG1-mut), significantly reduced luciferase activities in HCT-116 cells (Fig. 5d). In addition, RNA immunoprecipitation (RIP) assay results revealed that SNHG1 and miR-154-5p were significantly enriched in AGO2-containing micro-ribonucleoprotein complexes, suggesting that the AGO2 protein bound to SNHG1 and miR-154-5p directly in colorectal cancer cells (Fig. 5e and Additional file 7: Figure S3a). RNA pull-down assays also confirmed that SNHG1 could directly bind with AGO2 in colorectal cancer cells (Fig. 5f and Additional file 7: Figure S3b). Besides, we found SNHG1 overexpression reduced miR-154-5p expression, and knockdown of SNHG1 significantly increased miR-154-5p expression (Additional file 7: Figure S3c and Figure S3d). Above all, miR-154-5p overexpression weakened the proliferation promotion effect of SNHG1 overexpression, and miR-154-5p down-regulation rescued the growth inhibition caused by SNHG1 knockdown (Fig. 5g, h, Additional file 7: Figure S3e and Additional file 7: Figure S3f). Finally, correlation analysis revealed that SNHG1 expression is negatively associated with miR-154-5p in 30 colorectal cancer tissues (*P = 0.008*; Fig. 5i). The effects of miR-154-5p mimics and inhibitors were validated by qRT-PCR (Additional file 7: Figure S3 h). Altogether, our results indicated that SNHG1 acts as molecular sponge for miR-154-5p, and the tumor-promoting effect of SNHG1 was partly dependent on sponging miR-154-5p.

### SNHG1 regulates the expression of the miR-154-5p target gene, CCND2

**Fig. 6:**
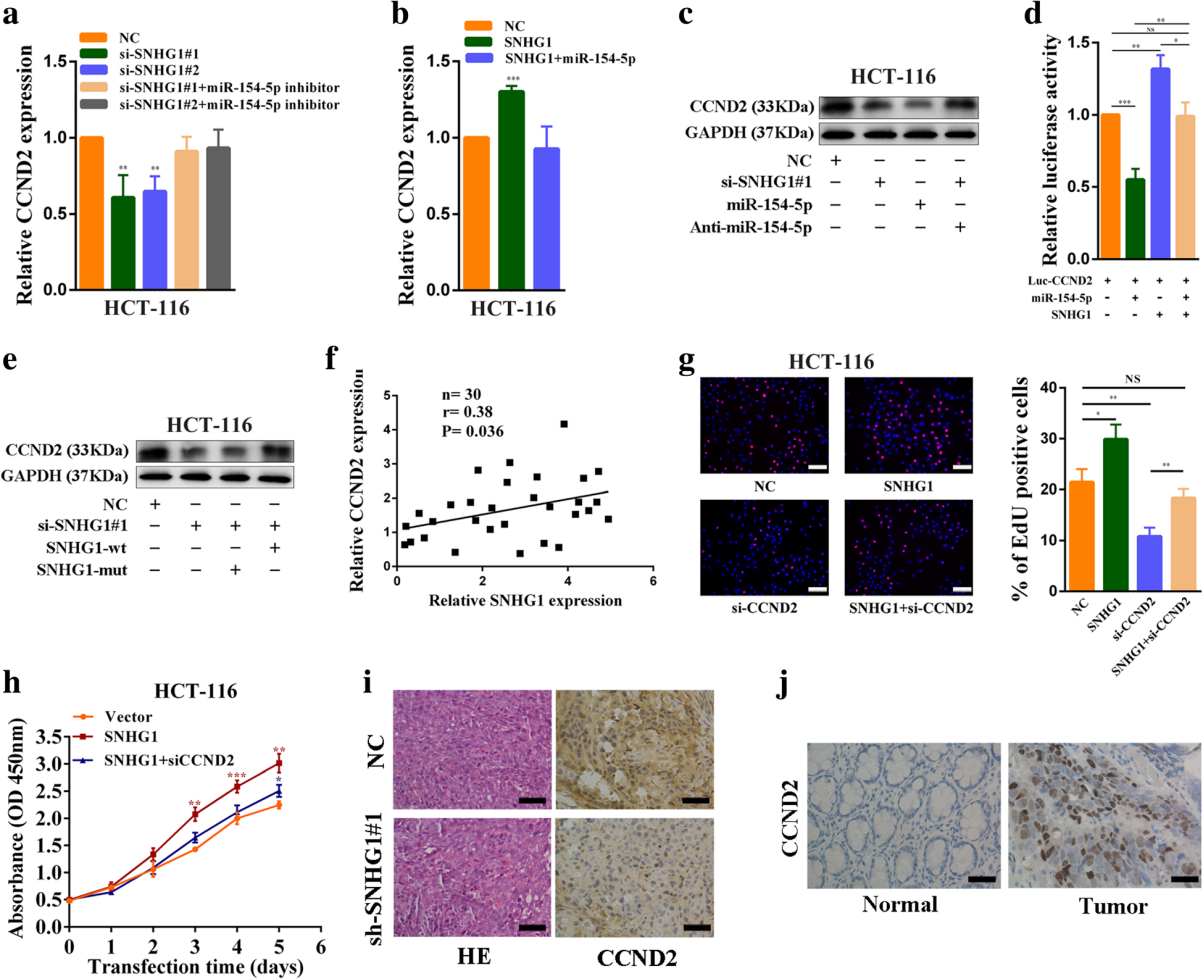
SNHG1 regulates expression of the miR-154-5p target gene, CCND2. **a** CCND2 expression was detected by qRT-PCR in SNHG1 siRNAs transfected or SNHG1 siRNAs and miR-154-5p inhibitors co-transfected HCT-116 cells. **b** CCND2 expression was detected by qRT-PCR in SNHG1 vector transfected or SNHG1 vector and miR-154-5p mimics co-transfected HCT-116 cells. **c** Western blot analyses of CCND2 expression after knockdown of SNHG1, overexpression of miR-154-5p or knockdown of SNHG1 + inhibition of miR-154-5p in HCT-116 cells. **d** Dual luciferase reporter assays demonstrated that miR-154-5p overexpression reduced Luc-CCND2 luciferase activity and SNHG1 overexpression abolished miR-154-5p induced reductions in luciferase activity in HCT-116 cells. **e** CCND2 expression was measured by western blot after silencing of endogenous SNHG1 and transfection with either SNHG1-mut vector, which contains mutations at the putative miR-154-5p binding site, or SNHG1 vector in HCT-116 cells. **f** The correlation between CCND2 and SNHG1 expression analyzed in 30 paired colorectal cancer samples (*n* = 30, r = 0.38, *P* = 0.036). **g** EdU assays demonstrated HCT-116 cells proliferation rates after knockdown of SNHG1, knockdown of CCND2 or both knockdown of SNHG1and CCND2. **h** CCK-8 assays demonstrated that CCND2 knockdown could reverse growth promotion caused by SNHG1 overexpression in HCT-116 cells. **i** Immunohistochemistry analysis of CCND2 protein levels in tumor tissues formed from SNHG1 knockdown or control cells. **j** Detection of CCND2 protein levels in colorectal cancer and normal tissues by IHC. Scale bar = 50 μm. *P < 0.05, **P < 0.01 and ***P < 0.001

Some studies demonstrated that miR-154-5p could act as a tumor suppressor gene through targeting CCND2, STAG2, E2F5, HMGA2 and TLR2 [23–27]. We then investigated the targets of SNHG1 ceRNA. Using expression analyses, we found that when SNHG1 was silenced, CCND2 but not STAG2, E2F5, HMGA2 or TLR2 was downregulated in HCT-116 cells (Additional file 8: Figure S4a). Besides, inhibition of miR-154-5p in SNHG1-downregulated cells reversed the reduction in CCND2 and overexpression of miR-154-5p abolished CCND2 increase in SNHG1-upregulated cells (Fig. 6a, b, Additional file 8: Figure S4b and Additional file 8: Figure S4c). Western blot assays also showed that inhibition of miR-154-5p could rescue CCND2 protein level decrease induced by SNHG1 knockdown (Fig. 6c and Additional file 8: Figure S4d). Then, we constructed luciferase reporter vectors Luc-CCND2 containing the 3′-untranslated region (3’-UTR) of CCND2 (Additional file 8: Figure S4e). As shown in Fig. 6d, miR-154-5p overexpression reduced Luc-CCND2 luciferase activity significantly in HCT-116 cells, indicating that CCND2 was a direct target of miR-154-5p. Then, Luc-CCND2 plasmids were co-transfected with the SNHG1 plasmids and miR-154-5p mimics. MiR-154-5p induced a reduction in luciferase activity that was completely abolished by SNHG1 overexpression. We next examined whether SNHG1 regulates CCND2 expression by putative miR-154-5p binding sequences. We silenced endogenous SNHG1 and then transfected cells with the SNHG1-mut vector, which contains mutations at the putative miR-154-5p binding site, or the SNHG1 vector. Following that, we measured CCND2 expression by western blot and found that SNHG1 knockdown downregulated CCND2 expression. In rescue experiments, transfection with wide type SNHG1 plasmids reversed the decrease in CCND2 expression, whereas transfection with mutant SNHG1 plasmids did not (Fig. 6e and Additional file 8: Figure S4f). Moreover, correlation analysis revealed that SNHG1 expression was positively associated with CCND2 expression in 30 colorectal cancer tissues (*P* = 0.036; Fig. 6f). A similar result was observed in colorectal cancer sequencing data in TCGA database (Additional file 8: Figure S4j).

Importantly, CCND2 silencing impaired SNHG1 overexpression mediated increases in colorectal cancer cells growth and proliferation (Fig. 6g, h, Additional file 8: Figure S4 g and Additional file 8: Figure S4 h). IHC assays showed that CCND2 expression was significantly decreased in xenografts formed from SNHG1 knockdown cells (Fig. 6i). Moreover, miR-154-5p was observed to be down-regulated in colorectal cancer tissues (Additional file 7: Figure S3 g), CCND2 was observed to be up-regulated in colorectal cancer tissues (Fig. 6j and Additional file 8: Figure S4i). Collectively, these results suggest that SNHG1 released CCND2 by sequestering endogenous miR-154-5p in colorectal cancers cells.

### SNHG1 is required for the epigenetic repression of KLF2 and CDKN2B by interacting with PRC2

**Fig. 7:**
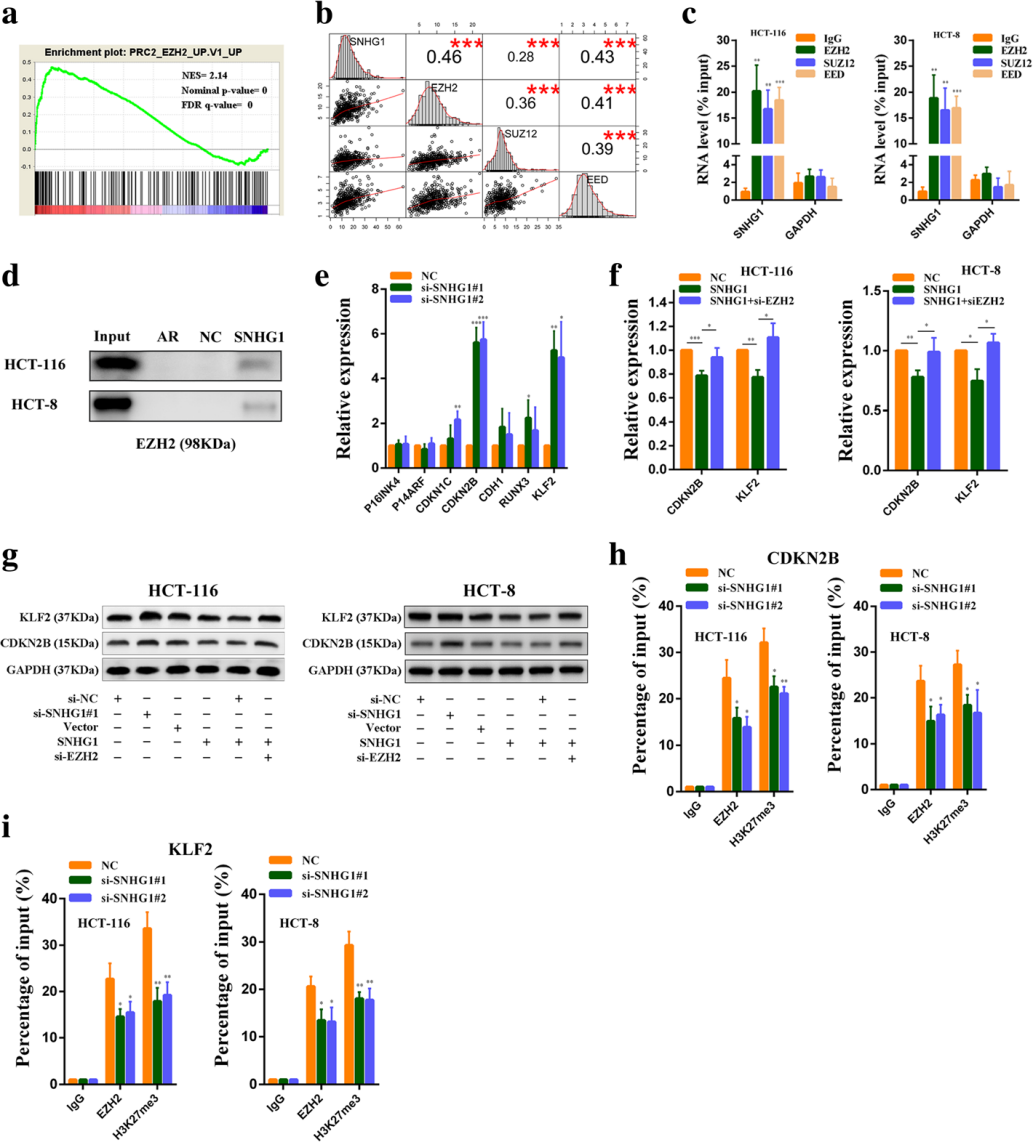
SNHG1 participates in epigenetic repression of KLF2 and CDKN2B by interacting with PRC2. **a** GSEA showed a significant correlation between the SNHG1 and genes in PRC2 related pathway. **b** Scatter plot showing the expression relationship among SNHG1, EZH2, SUZ12 and EED in colorectal tumor tissues from TCGA database. The upper right squares show the Pearson correlation between each other. **c** RIPs experiments for EZH2, SUZ12 and EED were performed and the coprecipitated RNA was subjected to qRT-PCR for SNHG1. GAPDH was employed as a negative control. **d** RNA pull-down was used to examine the association of SNHG1 and EZH2. AR binding to HuR was used as a positive control. **e** PRC2 target genes expression was detected by qRT-PCR in SNHG1 siRNAs transfected HCT-116 cells. **f** CDKN2B and KLF2 expression was detected by qRT-PCR in SNHG1 vector transfected or SNHG1 vector and EZH2 siRNAs co-transfected CRC cells. **g** CDKN2B and KLF2 protein levels were detected by western blot in indicated conditions. **h** ChIP assays were performed to detect EZH2 and H3K27me3 occupancy in the CDKN2B promoter region. **i** ChIP assays were performed to detect EZH2 and H3K27me3 occupancy in the KLF2 promoter region. *P < 0.05, **P < 0.01 and ***P < 0.001

We next explored SNHG1 functions in the nucleus. GSEA results revealed a significant correlation between SNHG1 and genes in the PRC2 related pathway (Fig. 7a). Previous studies have demonstrated that some lncRNAs in the nucleus could participate in the recruitment of PRC2 to its target genes and influence their expression. Thus, we predicted the interaction probabilities of SNHG1 and proteins in PRC2 by RNA-protein interaction prediction software RPISeq [28]. SNHG1 exhibited a high possibility of interacting with EZH2, SUZ12 and EED (as the RF or SVM score > 0.5; Additional file 9: Figure S5a). Besides, through analyzing colorectal cancer RNA sequencing data in TCGA, we found SNHG1 expression was positively correlated with EZH2, SUZ12 and EED (Fig. 7b). These bioinformatics analyses indicated that SNHG1 might be involved in PRC2 mediated epigenetic repression. To verify our hypothesis, we next performed RNA immunoprecipitation assays with EZH2, SUZ12 and EED antibodies. As we expected, RIP results revealed that SNHG1 bound with EZH2, SUZ12 and EED in colorectal cancer cells (Fig. 7c). In addition, RNA pull-down assays also confirmed that SNHG1 could directly bound with EZH2 in colorectal cancer cells (Fig. 7d). We then explored whether SNHG1 silencing affected PRC2 mediated epigenetic repression. Previous studies have reported that tumor suppressor genes KLF2, RUNX3, CDH1, CDKN1C, CDKN2B, P14 and P16 were targets of PRC2 [29–31]. So, we detected their expression in SNHG1 knockdown HCT-116 cells by qRT-PCR. As shown in Fig. 7e, downregulation of SNHG1 significantly increased KLF2 and CDKN2B expression compared with the control cells. Hence, we selected KLF2 and CDKN2B for further study. Next, we found that upregulation of SNHG1 could reduce KLF2 and CDKN2B expression, whereas EZH2 knockdown abolished these effects (Fig. 7f). Consistent with PCR results, western blot analysis revealed that SNHG1 silencing increased KLF2 and CDKN2B protein levels, SNHG1 overexpression reduced KLF2 and CDKN2B protein levels, and the decrease could be impaired by EZH2 knockdown (Fig. 7g). We also found that SNHG1 downregulation did not affect EZH2 expression (Additional file 9: Figure S5b). To further address whether SNHG1 was involved in transcriptional repression through enrichment of H3K27me3 to the promoter regions of KLF2 and CDKN2B, we conducted ChIP assays. We found that SNHG1 knockdown decreased EZH2 binding ability to their promoters. Similarly, PRC2 enrichment induced H3K27me3 modifications also reduced in their promoter regions after SNHG1 knockdown (Fig. 7h and Fig. 7i). Finally, we observed a negative correlation between SNHG1 and KLF2 or CDKN2B expression in colorectal cancer tissues by analyzing RNA sequencing data from TCGA database (Additional file 9: Figure S5e and Figure S5f). These data indicate that SNHG1 contributes to the epigenetic repression of KLF2 and CDKB2B through binding to EZH2.

### SNHG1 promotes colorectal cancer cell growth in part by regulating KLF2 and CDKN2B expression

**Fig. 8:**
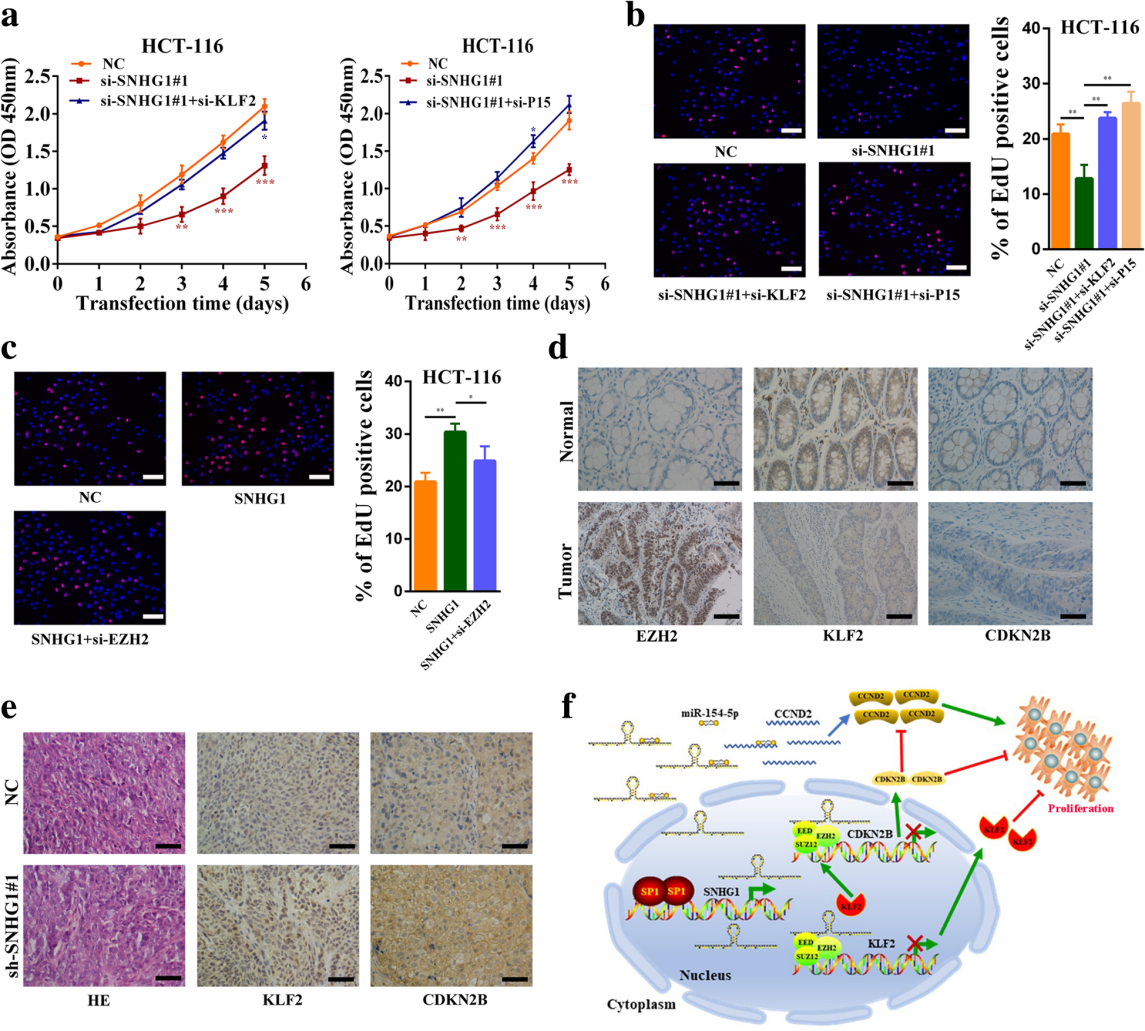
SNHG 1 promotes colorectal cancer progression partly by regulating KLF2 and CDKN2B expression. **a** Left panel, CCK-8 assays demonstrated that silence of SNHG1 inhibited cancer cell growth. KLF2 knockdown could rescue growth inhibition caused by SNHG1 knockdown in HCT-116 cells. Right panel, CCK-8 assays demonstrated that silence of SNHG1 inhibited cancer cell growth. CDKN2B (P15) knockdown could rescue growth inhibition caused by SNHG1 knockdown in HCT-116 cells. **b** EdU assays showed that SNHG1 knockdown inhibited cancer cell proliferation. Co-transfecting KLF2 or CDKN2B siRNAs with SNHG1 siRNAs reversed the decreased proliferation rates in HCT-116 cells. **c** EdU assays showed that EZH2 knockdown could inhibit proliferation promotion caused by SNHG1 overexpression in HCT-116 cells. **d** Immunohistochemistry analysis of EZH2, KLF2 and CDKN2B protein levels in colorectal cancer and normal tissues. **e** Immunohistochemistry analysis of KLF2 and CDKN2B protein levels in tumor tissues formed from SNHG1 knockdown or control cells. **f** Schematic of the proposed mechanism of SNHG1 in colorectal cancer cells. In the cytoplasm, SNHG1 acts as a ceRNA to sponge miR-154-5p and upregulated the expression of CCND2 (CyclinD2). In the nucleus, SNHG1 is involved in PRC2 mediated epigenetic repression of KLF2 and CDKN2B (P15). KLF2 is also an upstream regulatory factor of CDKN2B. Besides, CDKN2B is a well-studied inhibitor of CCND2. Downstream genes of SNHG1 formed a regulatory network to regulate growth of colorectal cancer. Scale bar = 50 μm. *P < 0.05, **P < 0.01 and ***P < 0.001

To investigate whether KLF2 and CDKN2B were involved in the SNHG1 induced promotion of colorectal cancer cell growth, we performed rescue assays. CCK-8 results revealed that silencing of KLF2 or CDKN2B (P15) partially abolished SNHG1 knockdown induced growth arrest of colorectal cancer cells (Fig. 8a and Additional file 10: Figure S6a). Similarly, EdU results revealed that silencing of KLF2 and CDKN2B could partially rescue SNHG1 knockdown induced reductions in cancer cell proliferative capacity (Fig. 8b and Additional file 10: Figure S6b). Furthermore, EZH2 silencing partially impaired SNHG1 overexpression mediated proliferative capacity increase of colorectal cancer cells (Fig. 8c and Additional file 10: Figure S6c). In addition, immunohistochemistry analyses revealed that EZH2 was upregulated in colorectal tumor tissues, whereas KLF2 and CDKN2B expression was reduced in cancer tissues (Fig. 8d). Similar results were observed in RNA sequencing data (Additional file 9: Figure S5c and Figure S5d). Moreover, IHC results demonstrated that KLF2 and CDKN2B were increased in xenograft tissues formed from SNHG1 knockdown cells (Fig. 8e). These data suggest that SNHG1 increases colorectal cancer cell growth partly depends on regulation of KLF2 and CDKN2B expression.

## Discussion

The key finding of this study is that SNHG1 plays a vital role in colorectal cancer progression. The results of our study demonstrated that SNHG1 was significantly upregulated in human colorectal cancer tissues, and its high expression was correlated with poor prognosis. Functional studies revealed tumorigenic roles of SNHG1 in promoting cell growth and cell cycle progression. Mechanistically, we found that SNHG1 increased CCND2 expression via its sponge activity of miR-154-5p. In addition, SNHG1 could regulate KLF2 and CDKN2B expression by interactions with Polycomb Repressive Complex 2. These results indicate that SNHG1 acts as an oncogene in colorectal cancer.

With the development of sequencing technology, many non-coding transcripts have been discovered. Among them, lncRNAs have attracted more and more attention gieven their wide range of functions. In tumors, various lncRNAs are abnormally expressed and some of them have been identified to have multiple effects in cancer initiation, progression and metastasis [7, 32]. SNHG1 belongs to a family of non-coding RNAs that hosting snoRNAs. Recent studies have demonstrated that its abnormal expression is associated with numerous diseases [33–36]. In colorectal cancer, Sun et al. reported that decreased SNHG1 expression inhibited colorectal carcinoma tumor genesis and Yang et al. reported that SNHG1 could promote colorectal cancer progression through the Wnt/β-catenin signaling pathway [37, 38]. Here, we revealed novel biological effects of SNHG1 in colorectal cancer.

Based on previous studies, many transcription factors are overexpressed in cancer tissues and several induce the up-regulation of lncRNAs. To identify the reason for high SNHG1 expression in colorectal cancer, we analyzed potential transcription factors binding sites in the SNHG1 promoter region. Combined with ChIP-Seq analyses results, we focused on the proto-oncogenic transcription factor SP1 and proved it could directly regulate SNHG1 expression in CRC.

To further explore the underlying molecular mechanisms by which SNHG1 regulated downstream effectors in colorectal cancer, we firstly identified its localization in cancer cells given that lncRNA functions are dependent on its subcellular localization. Nuclear lncRNAs can affect chromatin structure and gene transcription through recruiting related modification enzymes or transcription factors. Cytosolic lncRNAs can modulate mRNA stability, protein localization and act as microRNA sponge [21]. We found that SNHG1 was expressed both in the cytoplasm and in the nucleus. We provided the evidence that SNHG1 acted as a ceRNA for miR-154-5p in the cytoplasm and sponged miR-154-5p to release its inhibition of CCND2. MiR-154-5p was reported to be a tumor suppressor gene in various cancers [23–27]. CCND2 is a target gene of miR-154-5p and plays critical roles in cell cycle progression. CCND2 is upregulated in tumors and induce pRb inactivation and E2F-mediated upregulation of genes required for S phase entry [39, 40]. In this study, we found SNHG1 expression was positively related to CCND2 expression and SNHG1 could regulate CCND2 expression as ceRNAs.

Polycomb repressive complex-2 is a histone methyltransferase involved in epigenetic silencing during cancer development. To data, numerous PRC2 target genes have been identified. Given that PRC2 mostly acts as an oncogenic factor in cancers, many PRC2 targets identified in cancer are tumor suppressor genes [29]. The E-cadherin (CDH1) is regulated by PRC2, and its suppression is critical for Epithelial-Mesenchymal Transition (EMT) and cancer metastasis. Other critical targets of PRC2 in multiple cancers are the INK4B-ARF-INK4A tumor suppressor locus, the downregulation of these genes is critical for cancer cell proliferation. Besides, multiple tumor suppressor genes are involved in PRC2-mediated cancer progression. These target genes include CDKN1C, RUNX3 and KLF2 [29, 30]. Recently, the role of PRC2 and its interaction with various lncRNAs have attracted considerable interest. This excitement is primarily derived from the finding that the Xist and HOTAIR can silence gene expression by physically interacting with the PRC2 complex, and perturbing PRC2 localization at their target sites [41]. Here, we reported that SNHG1 could directly interact with EZH2, and participate in PRC2-mediated epigenetic silencing of KLF2 and CDKN2B. As a member of the Kruppel-like factor family, KLF2 is down-regulated and possesses tumor-suppressor features such as inhibition of cell proliferation and enhancement of DNA-damage-associated apoptosis in many cancers [42]. Some evidences showed that EZH2 could silence KLF2 expression and inhibit the tumor-suppressor features of KLF2 [13, 43]. CDKN2B, one of the two cyclin-dependent kinase inhibitors encoded by the INK4b-ARF-INK4a locus. It is a well-established tumor suppressor gene which can form a complex with CDK4 or CDK6 and prevents the activation of the cyclin dependent kinase to inhibit cell cycle progression. The INK4b-ARF-INK4a locus is believed to be regulated by Polycomb repressive complexes, and CDKN2B expression is frequently down-regulated in cancers [44, 45]. Interestingly, as a cyclin-dependent kinase inhibitor, CDKN2B is an upstream regulatory gene of CCND2 [46]. We think SNHG1 knockdown induced reductions in CCND2 may also be partly due to the CDKN2B upregulation. In addition, KLF2 acts as a tumor suppressor gene in part by regulating the expression of CDKN2B [43, 47]. Thus, the downstream genes of SNHG1 formed a regulatory network to regulate the initiation and progression of colorectal cancer (Fig. 8f).

## Conclusions

Taken together, the results of this study indicate that SNHG1 functions as an oncogene in colorectal cancer. High SNHG1 expression is associated with tumor progression and poor prognosis. SNHG1 promotes colorectal cancer cell growth through epigenetic silencing of KLF2 and CDKN2B in the nucleus. In the cytoplasm, SNHG1 acts as a sponge for miR-154-5p to weaken the suppressive effect of miR-154-5p on CCND2, thus facilitating cell proliferation. Our study reveals that SNHG1 can display diverse regulatory mechanisms in different subcellular locations, and downstream factors of SNHG1 can form a network to regulate colorectal cancer cell growth. Our results suggest a strategy for targeting SNHG1 as a potential biomarker and a therapeutic target in colorectal cancer patients.

## Additional files

Additional file 1: **Table S1.** SiRNAs and sh-RNAs sequence (DOCX 15 kb)

Additional file 2: **Table S2.** The list of primers (DOCX 16 kb)

Additional file 3: **Table S3.** Information of antibodies (DOCX 16 kb)

Additional file 4: Supplementary materials and methods. (DOCX 18 kb)

Additional file 5: **Figure S1.** SNHG1 is up-regulated in cancers, related to Fig. 1. (a) The SNHG1 expression in different tumor stages of colorectal cancer in TCGA cohort. (b) Kaplan-Meier survival analysis of CRC patients’ overall survival based on SNHG1 expression in TCGA database (*n* = 423, *P* = 0.099). (c) Exact copy numbers of SNHG1 transcript in colorectal cancer cell lines (DLD-1, HCT-116, HT-29, SW-620, HCT-8 and SW-480) and normal colorectal epithelial cells FHC were measured by using standard curve method. (d) Analyses of mean SNHG1 expression levels in bladder urothelial carcinoma (BLCA), breast invasive carcinoma (BRCA), head and neck squamous cell carcinoma (HNSC), kidney chromophobe (KICH), kidney renal clear cell carcinoma (KIRC), lung adenocarcinoma (LUAD), thyroid carcinoma (THCA), and uterine corpus endometrioid carcinoma (UCEC) using TCGA sequencing data. (TIF 370 kb)

Additional file 6: **Figure S2.** SP1 is up-regulated in colorectal cancer and positively correlated with SNHG1, related to Fig. 2. (a) Detection of SP1 protein levels in colorectal cancer and normal tissues by IHC. (b) Western blot analyses of SP1 expression after knockdown of SP1 or overexpression of SP1. (c) The relation between SP1 and SNHG1 expression analyzed in colorectal cancer samples from TCGA cohort (*n* = 478, *r* = 0.202, *P* < 0.001). Scale bar = 50 μm. ***P < 0.001. (TIF 1055 kb)

Additional file 7: **Figure S3.** SNHG1 acts as a ceRNA for miR-154-5p, related to Fig. 5. (a) RNA immunoprecipitation with an anti-Ago2 antibody was used to assess endogenous Ago2 binding to RNA in HCT-8 cells, IgG was used as the control. SNHG1 and miR-154-5p levels were determined by qRT–PCR and presented as fold enrichment in Ago2 relative to input. RIP efficiency of Ago2 protein was detected by western blot. (b) RNA pull-down assays were used to examine the interaction of SNHG1 and Ago2 in HCT-8 cells. (c) MiR-154-5p expression was detected by qRT-PCR in SNHG1 siRNAs transfected HCT-116 and HCT-8 cells. (d) MiR-154-5p expression was detected by qRT-PCR in SNHG1 vectors transfected HCT-116 and HCT-8 cells. (e) CCK-8 assays demonstrated that SNHG1 silencing inhibited HCT-8 cell growth. MiR-154-5p down-regulation rescued growth inhibition caused by SNHG1 knockdown. (f) EdU assays revealed that SNHG1 overexpression promotes HCT-8 cell proliferation. Co-transfecting miR-154-5p mimics with the SNHG1 plasmid abolished the increased proliferation rates. (g) MiR-154-5p expression analyzed in colorectal cancer samples and normal samples from TCGA cohort. (h) MiR-154-5p expression was detected by qRT-PCR in HCT-116 and HCT-8 cells after miR-154-5p mimics or inhibitors transfection. Scale bar = 50 μm. *P < 0.05, **P < 0.01 and ***P < 0.001. (TIF 956 kb)

Additional file 8: **Figure S4.** SNHG1 regulates CCND2 expression by competitively binding miR-154-5p, related to Fig. 6. (a) STAG2, E2F5, HMGA2 and TLR2 expression was detected by qRT-PCR in SNHG1 siRNAs transfected HCT-116 cells. (b) CCND2 expression was detected by qRT-PCR in SNHG1 siRNAs transfected or SNHG1 siRNAs and miR-154-5p inhibitors co-transfected HCT-8 cells. (c) CCND2 expression was detected by qRT-PCR in SNHG1 vector transfected or SNHG1 vector and miR-154-5p mimics co-transfected HCT-8 cells. (d) Western blot analyses of CCND2 expression after knockdown of SNHG1, overexpression of miR-154-5p or knockdown of SNHG1 + inhibition of miR-154-5p in HCT-8 cells. (e) Sequence alignment of miR-154-5p and its predicted binding sites (green) in CCND2. Predicted miR-154-5p target sequence (blue) in CCND2 (Luc-CCND2-wt). (f) CCND2 expression was measured by western blot after silencing of endogenous SNHG1 and transfection with either SNHG1-mut vector, which contains mutations at the putative miR-154-5p binding site, or SNHG1 vector in HCT-8 cells. (g) EdU assays demonstrated HCT-8 cells proliferation rates after knockdown of SNHG1, knockdown of CCND2 or both knockdown of SNHG1and CCND2. (h) CCK-8 assays demonstrated that CCND2 knockdown could reverse growth promotion caused by SNHG1 overexpression in HCT-8 cells. (i) CCND2 expression analyzed in colorectal cancer samples and normal samples from TCGA cohort. (j) The relation between CCND2 and SNHG1 expression analyzed in colorectal cancer samples from TCGA cohort (n = 478, *r* = 0.21, *P* = 0.003). Scale bar = 50 μm. *P < 0.05 and **P < 0.01. (TIF 1059 kb)

Additional file 9: **Figure S5.** SNHG1 is involved in epigenetic repression of KLF2 and P15 by interacting with PRC2., related to Fig. 7. (a) Bioinformatics were used to predict this possibility of interaction of SNHG1 and a panel of proteins, including AGO2, EZH2, SUZ12 and EED. Predictions with probabilities > 0.5 were considered positive. RPISeq predictions are based on Random Forest (RF) or Support Vector Machine (SVM). (b) EZH2 expression was detected by qRT-PCR in SNHG1 siRNAs transfected HCT-116 and HCT-8 cells. (c) KLF2 expression was analyzed in colorectal cancer samples and normal samples from TCGA cohort. (d) CDKN2B expression was analyzed in colorectal cancer samples and normal samples from TCGA cohort. (e) The relation between KLF2 and SNHG1 expression analyzed in colorectal cancer samples from TCGA cohort (n = 478, *r* = − 0.24, *P* < 0.001). (f) The relation between CDKN2B and SNHG1 expression analyzed in colorectal cancer samples from TCGA cohort (n = 478, *r* = − 0.20, *P* < 0.001). *P < 0.05 and ***P < 0.001. (TIF 483 kb)

Additional file 10: **Figure S6.** SNHG1 promotes HCT-8 cell growth partly by regulating KLF2 and CDKN2B expression., related to Fig. 8. (a) Left panel, CCK-8 assays demonstrated that silence of SNHG1 inhibited cancer cell growth. KLF2 knockdown could rescue growth inhibition caused by SNHG1 knockdown in HCT-8 cells. Right panel, CCK-8 assays demonstrated that silence of SNHG1 inhibited cancer cell growth. CDKN2B (P15) knockdown could rescue growth inhibition caused by SNHG1 knockdown in HCT-8 cells. (b) EdU assays showed that SNHG1 knockdown inhibited cancer cell proliferation. Co-transfecting KLF2 or CDKN2B siRNAs with SNHG1 siRNAs reversed the decreased proliferation rates in HCT-8 cells. (c) EdU assays showed that EZH2 knockdown could inhibit proliferation promotion caused by SNHG1 overexpression in HCT-8 cells. Scale bar = 50 μm. *P < 0.05, **P < 0.01 and ***P < 0.001. (TIF 2205 kb)

## Abbreviations

3’-UTR: 3′-untranslated region
ANOVA: analysis of variance
AR: androgen receptor
CCND2: Cyclin D2
CDH1: E-cadherin
CDKN2B: Cyclin dependent kinase inhibitor 2B
ceRNAs: competing endogenous RNAs
ChIP: Chromatin immunoprecipitation
CI: Confidential interval
CRC: Colorectal cancer
DMEM: Dulbecco’s modified Eagle’s medium
EdU: Ethynyl deoxy Uridine
EMT: Epithelial-Mesenchymal Transition
ENCODE: Encyclopedia of DNA Elements (ENCODE)
FISH: Fluorescence in situ hybridization
GEO: Gene Expression Omnibus
GSEA: Gene Set Enrichment Analysis
H3K27me3: Histone H3 lysine 27 trimethylation
HE: Hematoxylin and eosin
HR: Hazard ratio
IHC: Immunohistochemistry
KLF2: Kruppel like factor 2
lncRNAs: Long noncoding RNAs
ncRNAs: Non-coding RNAs
PcG: Polycomb group
PRC2: Polycomb Repressive Complex 2
qRT-PCR: quantitative reverse transcription polymerase chain reaction
RIP: RNA immunoprecipitation
SD: Standard deviation
SNHG1: Small nucleolar RNA host gene 1
TCGA: The Cancer Genome Atlas
