# Supplementary material for: The long noncoding RNA SNHG1 regulates colorectal cancer cell growth through interactions with EZH2 and miR-154-5p
Source: Mol Cancer. 2018 Sep 28;17:141. doi: 10.1186/s12943-018-0894-x (PMC6162892; doi:10.1186/s12943-018-0894-x)
Supplement: Supplementary file 1 — Table S1. SiRNAs and sh-RNAs sequence (DOCX 15 kb) [file 12943_2018_894_MOESM1_ESM.docx]

**Table S1: SiRNAs and sh-RNAs sequence.**

| **siRNAs** |  |  |
| --- | --- | --- |
|  | **sense sequence** | **anti-sense sequence** |
| si-SP1#1 | 5'-CCUGGAGUGAUGCCUAAUATT-3' | 5'-UAUUAGGCAUCACUCCAGGTT-3' |
| si-SP1#2 | 5'-GUGCAAACCAACAGAUUAUTT-3' | 5'-AUAAUCUGUUGGUUUGCACTT-3' |
| si-SNHG1#1 | 5'-CCAGCAUCUCAUAAUCUAUtt-3' | 5'-AUAGAUUAUGAGAUGCUGGaa-3' |
| si-SNHG1#2 | 5'-CCUUCUCUCUAAAGCCCAAtt-3' | 5'-UUGGGCUUUAGAGAGAAGGtg-3' |
| si-CCND2 | 5'-GAUCAUAUUUAAAGAUCUUTT-3' | 5'-AAGAUCUUUAAAUAUGAUCTT-3' |
| si-KLF2 | 5'-CAAAAUGGUGCAAUAAUUUTT-3' | 5'-AAAUUAUUGCACCAUUUUGTT-3' |
| si-CDKN2B | 5'-GAAUAACCUUCCAUACAUUTT-3' | 5'-AAUGUAUGGAAGGUUAUUCTT-3' |
| si-EZH2 | 5'-CGGCUUCCCAAUAACAGUATT-3' | 5'-UACUGUUAUUGGGAAGCCGTT-3' |
| **shRNAs** |  |  |
| sh-SNHG1#1 | ggccagcaccttctctctaaactcgagtttagagagaaggtgctggccttttt | |
| sh-SNHG1#2 | ggtttgctgtgtatcacatttctcgagaaatgtgatacacagcaaaccttttt | |
