# Supplementary material for: The long noncoding RNA SNHG1 regulates colorectal cancer cell growth through interactions with EZH2 and miR-154-5p
Source: Mol Cancer. 2018 Sep 28;17:141. doi: 10.1186/s12943-018-0894-x (PMC6162892; doi:10.1186/s12943-018-0894-x)
Supplement: Supplementary file 2 — Table S2. The list of primers (DOCX 16 kb) [file 12943_2018_894_MOESM2_ESM.docx]

**Table S2: The list of primers.**

| **qPCR primers** | |  |
| --- | --- | --- |
|  | **Forward Primer** | **Reverse Primer** |
| SNHG1 | GCCAGCACCTTCTCTCTAAAGC | GTCCTCCAAGACAGATTCCATTTT |
| SP1 | TCCAGACCATTAACCTCAGTGC | TGTATTCCATCACCACCAGCC |
| CCND2 | TCCAAACTCAAAGAGACCAGC | TTCCACTTCAACTTCCCCAG |
| STAG2 | TCCTTCTGGTCCAAACCGAAT | ACCGACTGCATAGCACTCTTG |
| E2F5 | TCATTCAGGACCTATCCATGTGC | GTCACTGGAGTCAAGGACTGG |
| HMGA2 | ACCCAGGGGAAGACCCAAA | CCTCTTGGCCGTTTTTCTCCA |
| TLR2 | CCTCTCGGTGTCGGAATGTC | TCCCGCTCACTGTAAGAAACA |
| EZH2 | AATCAGAGTACATGCGACTGAGA | GCTGTATCCTTCGCTGTTTCC |
| P16INK4 | ATGGAGCCTTCGGCTGACT | GTAACTATTCGGTGCGTTGGG |
| P14ARF | GGGTTTTCGTGGTTCACATCC | CTAGACGCTGGCTCCTCAGTA |
| CDKN2C | GGGGACCTAGAGCAACTTACT | CAGCGCAGTCCTTCCAAAT |
| CDH1 | ATTTTTCCCTCGACACCCGAT | TCCCAGGCGTAGACCAAGA |
| KLF2 | AGAGGGTCTCCCTCGATGAC | TCTCACAAGGCATCACAAGC |
| CDKN2B | CTGGACCTGGTGGCTACG | ACATTGGAGTGAACGCATCG |
| RUNX3 | AGCACCACAAGCCACTTCAG | GGGAAGGAGCGGTCAAACTG |
| U6 | CTCGCTTCGGCAGCACA | AACGCTTCACGAATTTGCGT |
| GAPDH | GGGAGCCAAAAGGGTCATCA | TGATGGCATGGACTGTGGTC |
| **primers for ChIP** | |  |
|  | **Forward Primer** | **Reverse Primer** |
| SNHG1 | AAGTTCAGGTGGCGCTTTGTCTA | ACGATTAAATACCATGCCGCAGA |
| CDKN2B | TCTGGTAAGGGTGTGCTGTG | AAAACTCCTCTGTGGCATGTG |
| KLF2 | CCTCAGTTTCCCTGCACTTGAC | GAGATACAATCACACCACTAC |
