# Supplementary material for: The long noncoding RNA SNHG1 regulates colorectal cancer cell growth through interactions with EZH2 and miR-154-5p
Source: Mol Cancer. 2018 Sep 28;17:141. doi: 10.1186/s12943-018-0894-x (PMC6162892; doi:10.1186/s12943-018-0894-x)
Supplement: Supplementary file 3 — Table S3. Information of antibodies (DOCX 16 kb) [file 12943_2018_894_MOESM3_ESM.docx]

|  |  | **Western blot** | **ChIP** | **RIP** | **IHC** |
| --- | --- | --- | --- | --- | --- |
| Anti-KLF2 | abcam #ab203591 | 1:1000 |  |  | 1:200 |
| Anti-cyclinD2 | abcam #ab207604 | 1:1000 |  |  | 1:100 |
| Anti-p15 INK4b | abcam #ab53034 | 1:1000 |  |  | 1:100 |
| SP1(D4C3) Rabbit mAb | cell signaling technology #9389 |  | 1:100 |  | 1:200 |
| Tri-Methyl-Histone H3 (Lys27) (C36B11) Rabbit mAb | cell signaling technology #9733 |  | 1:100 |  |  |
| Ezh2 (D2C9) XP Rabbit mAb | cell signaling technology #5246 | 1:1000 | 1:100 | 1:100 | 1:50 |
| SUZ12(D39F6) XP Rabbit mAb | cell signaling technology #3737 |  |  | 1:100 |  |
| EED Antibody (3B12) | Santa Cruz #sc-293203 |  |  | 1:100 |  |
| HuR Monoclonal Antibodies (Mouse) | Thermo fisher | 1:200 |  |  |  |
| CDK4 (D9G3E) Rabbit mAb | cell signaling technology #12790 | 1:1000 |  |  |  |
| CDK6 (D4S8S) Rabbit mAb | cell signaling technology #13331 | 1:1000 |  |  |  |
| Cyclin D1 Mouse Monoclonal antibody | proteintech #60186-1-lg | 1:2000 |  |  |  |
| Bax (D2E11) Rabbit mAb | cell signaling technology #5023 | 1:1000 |  |  |  |
| Caspase-3 Antibody | abcam #ab9662 | 1:1000 |  |  |  |
| PARP (46D11) Rabbit mAb | cell signaling technology #9532 | 1:1000 |  |  |  |
| Argonaute 2 (C34C6) Rabbit mAb | cell signaling technology #2897 | 1:1000 |  | 1:100 |  |
| KI67 Rabbit Polyclonal antibody | proteintech #27309-1-AP |  |  |  | 1:4000 |
| GAPDH (D16H11) XP Rabbit mAb | cell signaling technology #5174 | 1:1000 |  |  |  |

**Table S3: Information of antibodies.**
