# Supplementary material for: The long noncoding RNA SNHG1 regulates colorectal cancer cell growth through interactions with EZH2 and miR-154-5p
Source: Mol Cancer. 2018 Sep 28;17:141. doi: 10.1186/s12943-018-0894-x (PMC6162892; doi:10.1186/s12943-018-0894-x)
Supplement: Supplementary file 4 — Supplementary materials and methods. (DOCX 18 kb) [file 12943_2018_894_MOESM4_ESM.docx]

**Supplementary materials and methods**

**Tissue samples and clinical data collection**

The 160 samples of CRC and 80 adjacent nontumor tissues were obtained from patients during operation at Affiliated Nanjing First Hospital of Nanjing Medical University (Nanjing, China). All collected tissue samples were immediately snap frozen in liquid nitrogen and stored at -80 °C until required. The patient characteristics were listed in Table 1. This study was approved by the ethics committee on Human Research of the Nanjing First Hospital and written informed consent was obtained from all patients.

**RNA isolation and quantitative reverse transcription polymerase chain reaction (****qRT-PCR)**

Total RNAs were isolated from tissues and cells by TRIzol (Invitrogen, USA) according to the protocol. qRT-PCR was performed using SYBR Green PCR Kit (Takara, Japan) on an ABI 7500 System. GAPDH was employed as a control for normalization when detected mRNA and lncRNA expression. MicroRNA detection was using miDETECT A Track Kit (RiboBio, China). The U6 small nuclear RNA was used as a control for normalization. Each experiment was repeated at least three times and the data were analyzed using the 2^ΔΔ^CT method. Transcriptional copy number was measured by using standard curve method and the exact copy numbers of SNHG1 transcript were calculated by relating the Ct value to standard curve. Primers are listed in Additional file 1: Table S1.

**Protein extraction and western blot**

Total proteins were extracted from cultured cells using cell lysis buffer. Then, the protein samples were loaded onto 10% sodium dodecyl sulfate polyacrylamide gel electrophoresis. The membranes were blocked with 5 % non-fat milk in Tris-buffered saline and incubated with a specific primary antibody and a secondary antibody. Protein expression was detected by enhanced chemiluminescence kit. Antibodies used in this study were listed in Additional file 2: Table S2.

**Flow cytometry**

After 24 hours transfection, HCT-116 and HCT-8 cells were firstly trypsinized and washed with cold PBS, then they were fixed in 70% ethanol for 24 hours and stained with Propidium Iodide (PI) for 30 minutes. Finally, the cells were analyzed by FACScan flow cytometer (BD, USA). The cell cycle data were analyzed by ModFit LT software (Veirty, USA). Cell apoptosis was analyzed using the Annexin V-FITC/ (PI) Apoptosis Detection Kit (BD, USA) according to the protocol. Cells were stained with FITC and PI and then analyzed using FACScan (BD, USA). The cell apoptosis data were analyzed by Flowjo software (Tree Star, USA).

**Cell growth and colony formation assays**

For CCK-8 assays, HCT-116 and HCT-8 cells were seeded into 96-well plates at the density of 1×10^3^ (cells/well), and the absorbance at 450nm was measured on days 1, 2, 3, 4 and 5 with 10 μl of CCK-8 solution treated. For cell colony formation assays, 24 hours after transfection, 500 HCT-116 and HCT-8 cells were incubated in 6-well plates at 37˚C, 5% CO_2_. Two weeks later, the cells were stained with crystal violet (0.2%) for 30 min and the colony numbers were counted.

**5-Ethynyl-20-deoxyuridine (EdU) incorporation assay**

HCT-116 and HCT-8 cells were seeded at a density of 5×10^3^ cells per well in 96-well plates and cultured overnight. The newly synthesized DNA of the cells was assessed by the EdU incorporation assays using a Cell-Light EdU DNA Cell Proliferation Kit (Ribobio, China), according to the manufacturer’s instructions. The EdU incorporation rate was expressed as the ratio of EdU positive cells (red cells) to total Hoechst33342 positive cells (blue cells).

**Subcellular fractionation location**

The separation of the nuclear and cytosolic fractions was using the PARIS Kit (Invitrogen, USA) according to the manufacturer’s instructions.

**Immunohistochemistry (IHC)**

IHC staining was performed using Dako Envision System (Dako, Carpinteria, CA) according to the manufacturer’s guidelines. The IHC-stained tissue sections were scored by two pathologists who were blinded to the clinical parameters, respectively. The percentage of immunostaining and the staining intensity (0, negative; 1+, weak; 2+, moderate; and 3+, strong) were recorded. An H-score was calculated using the following formula: [1 × (% cells 1+) + 2 × (% cells 2+) + 3 × (% cells 3+)] × 100. The maximum H-score would be 300, corresponding to 100% of cells with strong intensity.

Antibodies information was listed in Additional file 2: Table S2.
