# Supplementary figures and images for: The long noncoding RNA SNHG1 regulates colorectal cancer cell growth through interactions with EZH2 and miR-154-5p
Source: Mol Cancer. 2018 Sep 28;17:141. doi: 10.1186/s12943-018-0894-x (PMC6162892; doi:10.1186/s12943-018-0894-x)

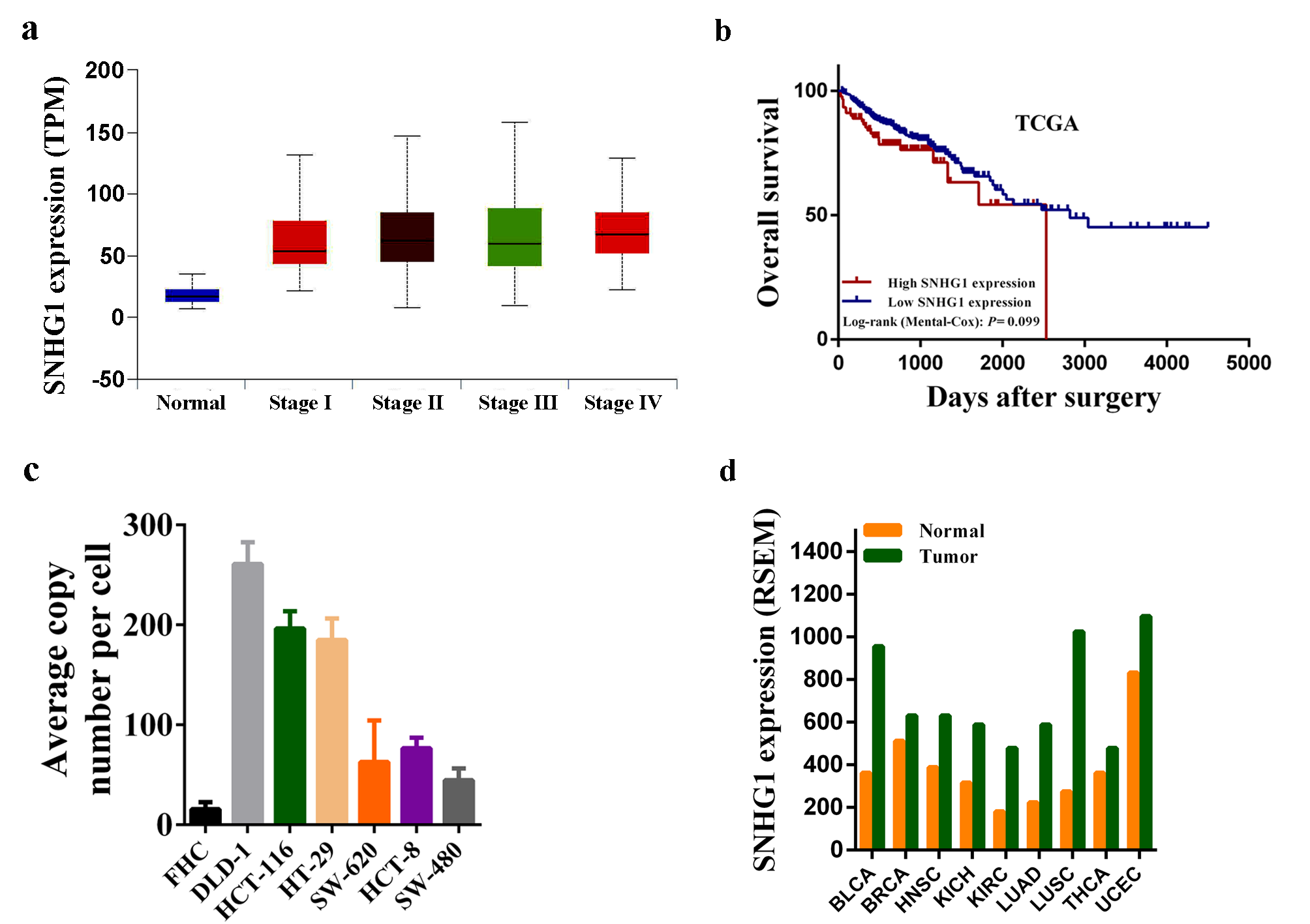

Supplement: Supplementary file 5 — Figure S1. SNHG1 is up-regulated in cancers, related to Fig. 1. (a) The SNHG1 expression in different tumor stages of colorectal cancer in TCGA cohort. (b) Kaplan-Meier survival analysis of CRC patients’ overall survival based on SNHG1 expression in TCGA database (n = 423, P = 0.099). (c) Exact copy numbers of SNHG1 transcript in colorectal cancer cell lines (DLD-1, HCT-116, HT-29, SW-620, HCT-8 and SW-480) and normal colorectal epithelial cells FHC were measured by using standard curve method. (d) Analyses of mean SNHG1 expression levels in bladder urothelial carcinoma (BLCA), breast invasive carcinoma (BRCA), head and neck squamous cell carcinoma (HNSC), kidney chromophobe (KICH), kidney renal clear cell carcinoma (KIRC), lung adenocarcinoma (LUAD), thyroid carcinoma (THCA), and uterine corpus endometrioid carcinoma (UCEC) using TCGA sequencing data. (TIF 370 kb) [file 12943_2018_894_MOESM5_ESM.tif]

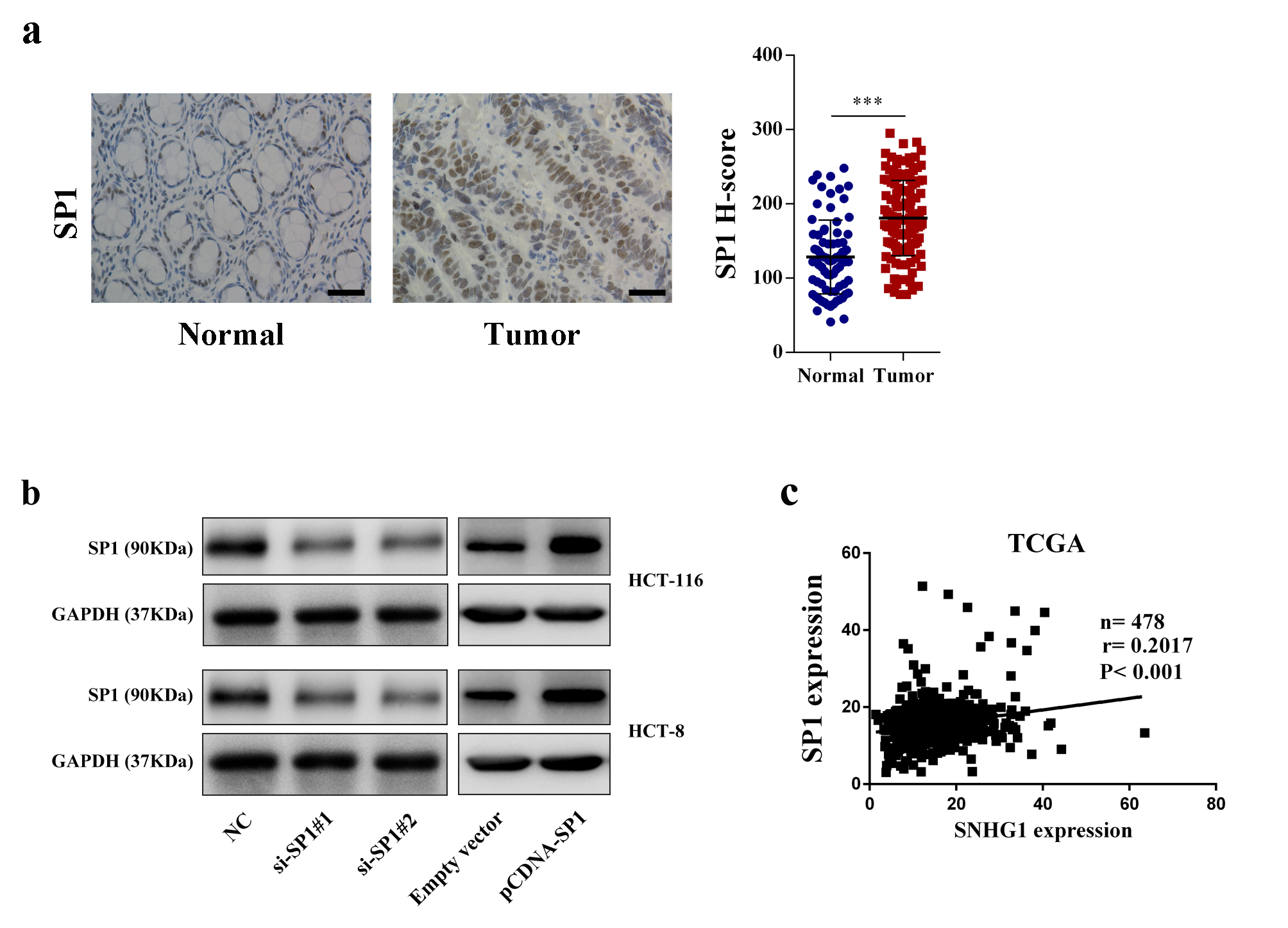

Supplement: Supplementary file 6 — Figure S2. SP1 is up-regulated in colorectal cancer and positively correlated with SNHG1, related to Fig. 2. (a) Detection of SP1 protein levels in colorectal cancer and normal tissues by IHC. (b) Western blot analyses of SP1 expression after knockdown of SP1 or overexpression of SP1. (c) The relation between SP1 and SNHG1 expression analyzed in colorectal cancer samples from TCGA cohort (n = 478, r = 0.202, P < 0.001). Scale bar = 50 μm. ***P < 0.001. (TIF 1055 kb) [file 12943_2018_894_MOESM6_ESM.tif]

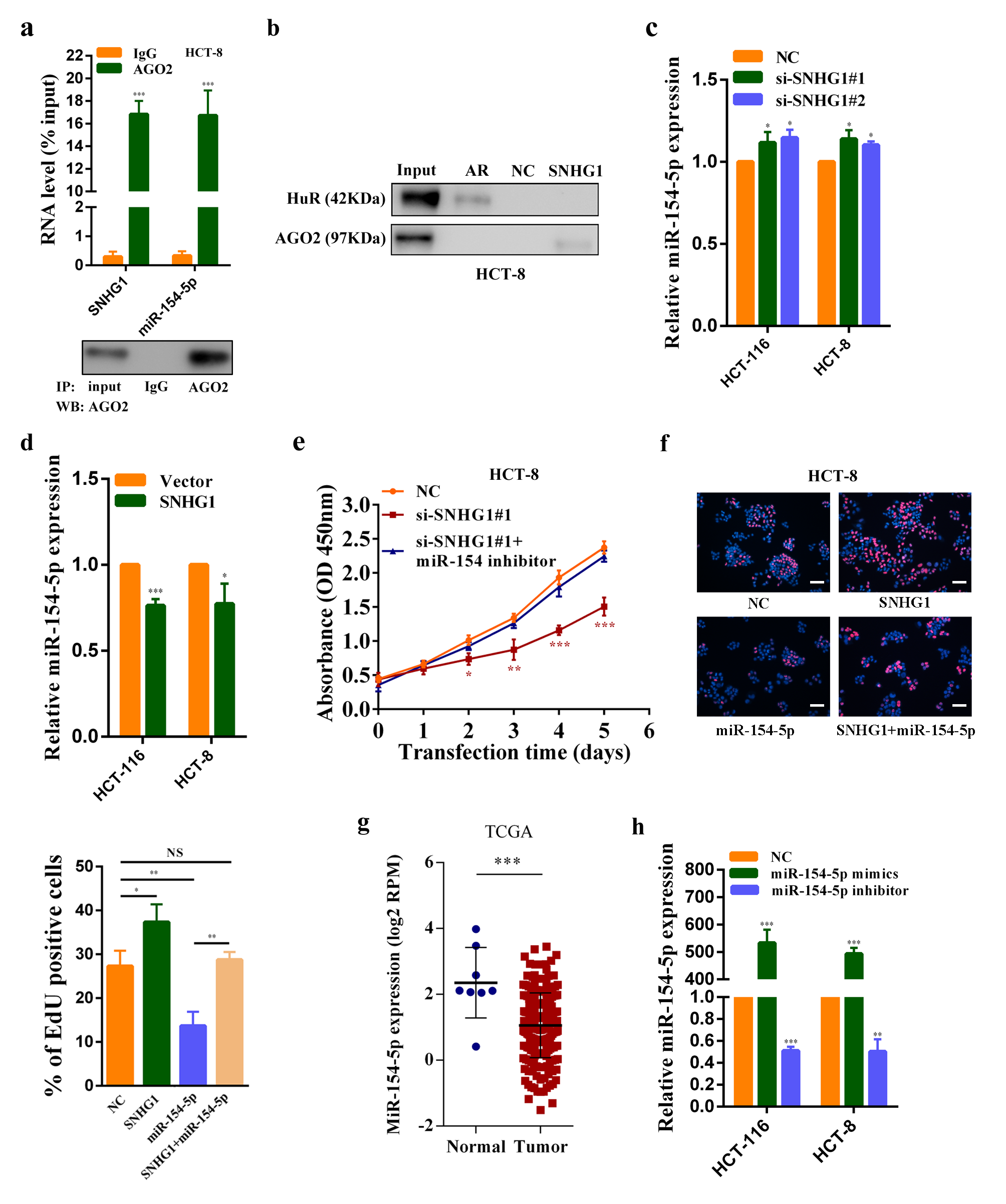

Supplement: Supplementary file 7 — Figure S3. SNHG1 acts as a ceRNA for miR-154-5p, related to Fig. 5. (a) RNA immunoprecipitation with an anti-Ago2 antibody was used to assess endogenous Ago2 binding to RNA in HCT-8 cells, IgG was used as the control. SNHG1 and miR-154-5p levels were determined by qRT–PCR and presented as fold enrichment in Ago2 relative to input. RIP efficiency of Ago2 protein was detected by western blot. (b) RNA pull-down assays were used to examine the interaction of SNHG1 and Ago2 in HCT-8 cells. (c) MiR-154-5p expression was detected by qRT-PCR in SNHG1 siRNAs transfected HCT-116 and HCT-8 cells. (d) MiR-154-5p expression was detected by qRT-PCR in SNHG1 vectors transfected HCT-116 and HCT-8 cells. (e) CCK-8 assays demonstrated that SNHG1 silencing inhibited HCT-8 cell growth. MiR-154-5p down-regulation rescued growth inhibition caused by SNHG1 knockdown. (f) EdU assays revealed that SNHG1 overexpression promotes HCT-8 cell proliferation. Co-transfecting miR-154-5p mimics with the SNHG1 plasmid abolished the increased proliferation rates. (g) MiR-154-5p expression analyzed in colorectal cancer samples and normal samples from TCGA cohort. (h) MiR-154-5p expression was detected by qRT-PCR in HCT-116 and HCT-8 cells after miR-154-5p mimics or inhibitors transfection. Scale bar = 50 μm. *P < 0.05, **P < 0.01 and ***P < 0.001. (TIF 956 kb) [file 12943_2018_894_MOESM7_ESM.tif]

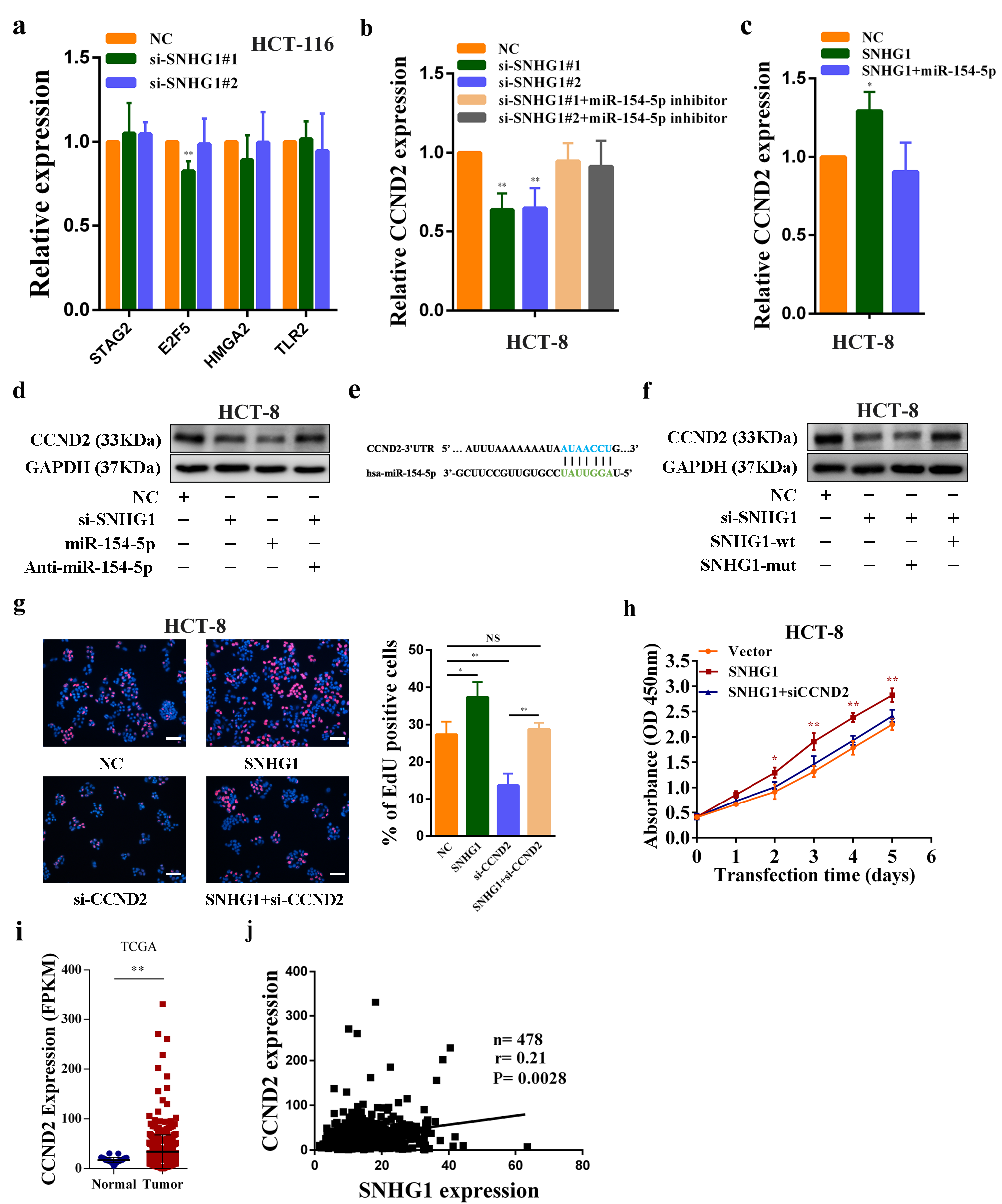

Supplement: Supplementary file 8 — Figure S4. SNHG1 regulates CCND2 expression by competitively binding miR-154-5p, related to Fig. 6. (a) STAG2, E2F5, HMGA2 and TLR2 expression was detected by qRT-PCR in SNHG1 siRNAs transfected HCT-116 cells. (b) CCND2 expression was detected by qRT-PCR in SNHG1 siRNAs transfected or SNHG1 siRNAs and miR-154-5p inhibitors co-transfected HCT-8 cells. (c) CCND2 expression was detected by qRT-PCR in SNHG1 vector transfected or SNHG1 vector and miR-154-5p mimics co-transfected HCT-8 cells. (d) Western blot analyses of CCND2 expression after knockdown of SNHG1, overexpression of miR-154-5p or knockdown of SNHG1 + inhibition of miR-154-5p in HCT-8 cells. (e) Sequence alignment of miR-154-5p and its predicted binding sites (green) in CCND2. Predicted miR-154-5p target sequence (blue) in CCND2 (Luc-CCND2-wt). (f) CCND2 expression was measured by western blot after silencing of endogenous SNHG1 and transfection with either SNHG1-mut vector, which contains mutations at the putative miR-154-5p binding site, or SNHG1 vector in HCT-8 cells. (g) EdU assays demonstrated HCT-8 cells proliferation rates after knockdown of SNHG1, knockdown of CCND2 or both knockdown of SNHG1and CCND2. (h) CCK-8 assays demonstrated that CCND2 knockdown could reverse growth promotion caused by SNHG1 overexpression in HCT-8 cells. (i) CCND2 expression analyzed in colorectal cancer samples and normal samples from TCGA cohort. (j) The relation between CCND2 and SNHG1 expression analyzed in colorectal cancer samples from TCGA cohort (n = 478, r = 0.21, P = 0.003). Scale bar = 50 μm. *P < 0.05 and **P < 0.01. (TIF 1059 kb) [file 12943_2018_894_MOESM8_ESM.tif]

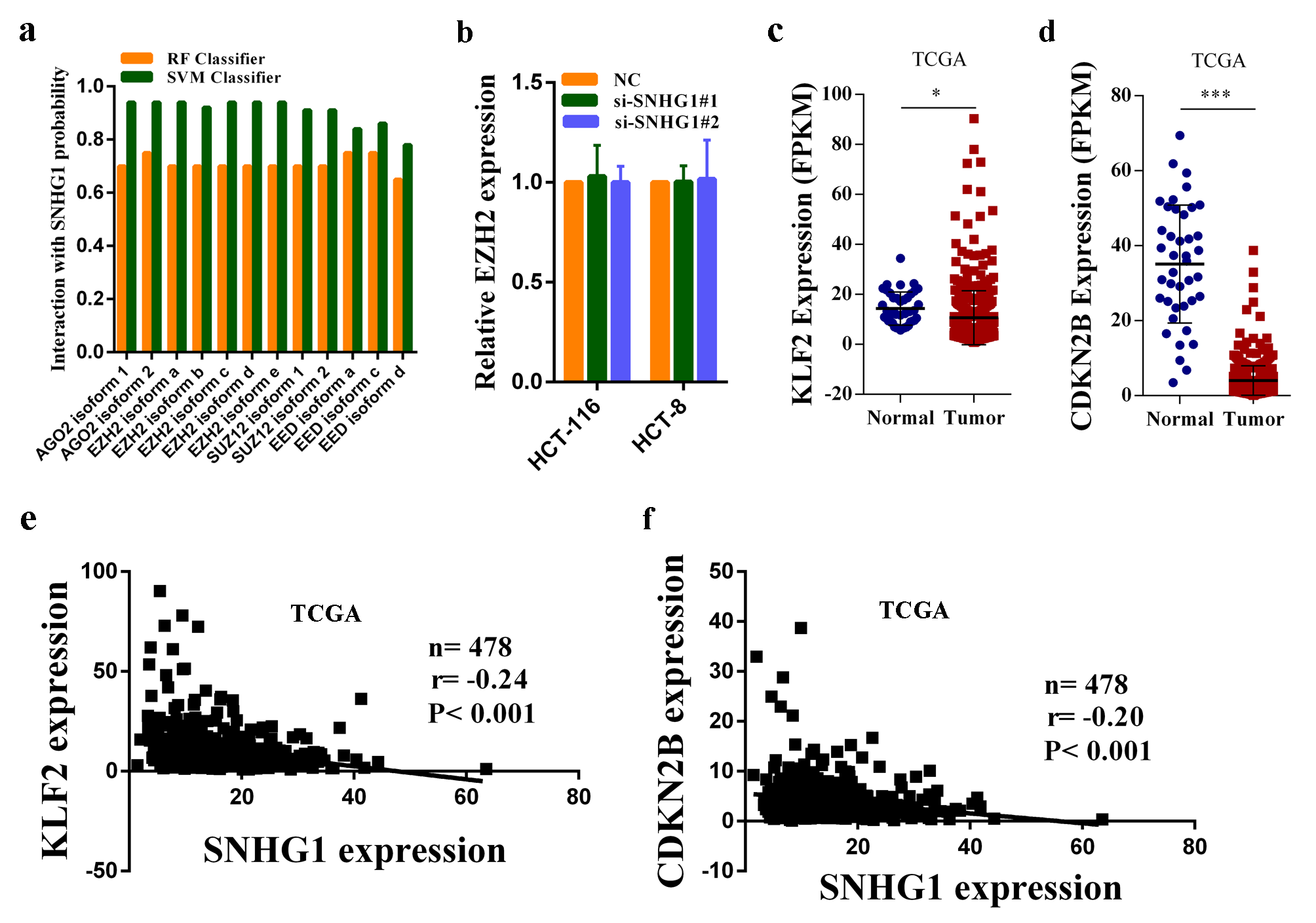

Supplement: Supplementary file 9 — Figure S5. SNHG1 is involved in epigenetic repression of KLF2 and P15 by interacting with PRC2., related to Fig. 7. (a) Bioinformatics were used to predict this possibility of interaction of SNHG1 and a panel of proteins, including AGO2, EZH2, SUZ12 and EED. Predictions with probabilities > 0.5 were considered positive. RPISeq predictions are based on Random Forest (RF) or Support Vector Machine (SVM). (b) EZH2 expression was detected by qRT-PCR in SNHG1 siRNAs transfected HCT-116 and HCT-8 cells. (c) KLF2 expression was analyzed in colorectal cancer samples and normal samples from TCGA cohort. (d) CDKN2B expression was analyzed in colorectal cancer samples and normal samples from TCGA cohort. (e) The relation between KLF2 and SNHG1 expression analyzed in colorectal cancer samples from TCGA cohort (n = 478, r = − 0.24, P < 0.001). (f) The relation between CDKN2B and SNHG1 expression analyzed in colorectal cancer samples from TCGA cohort (n = 478, r = − 0.20, P < 0.001). *P < 0.05 and ***P < 0.001. (TIF 483 kb) [file 12943_2018_894_MOESM9_ESM.tif]

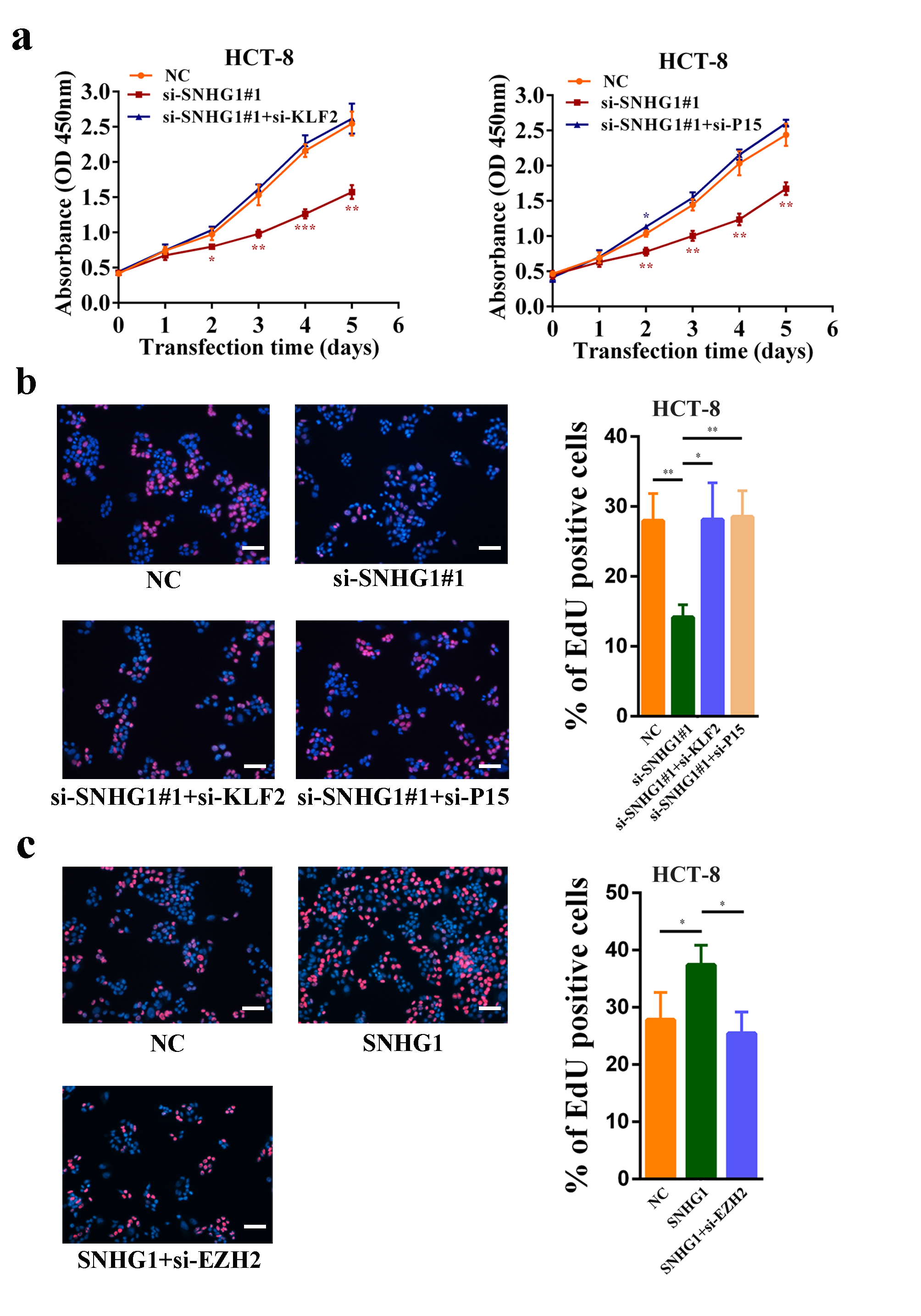

Supplement: Supplementary file 10 — Figure S6. SNHG1 promotes HCT-8 cell growth partly by regulating KLF2 and CDKN2B expression., related to Fig. 8. (a) Left panel, CCK-8 assays demonstrated that silence of SNHG1 inhibited cancer cell growth. KLF2 knockdown could rescue growth inhibition caused by SNHG1 knockdown in HCT-8 cells. Right panel, CCK-8 assays demonstrated that silence of SNHG1 inhibited cancer cell growth. CDKN2B (P15) knockdown could rescue growth inhibition caused by SNHG1 knockdown in HCT-8 cells. (b) EdU assays showed that SNHG1 knockdown inhibited cancer cell proliferation. Co-transfecting KLF2 or CDKN2B siRNAs with SNHG1 siRNAs reversed the decreased proliferation rates in HCT-8 cells. (c) EdU assays showed that EZH2 knockdown could inhibit proliferation promotion caused by SNHG1 overexpression in HCT-8 cells. Scale bar = 50 μm. *P < 0.05, **P < 0.01 and ***P < 0.001. (TIF 2205 kb) [file 12943_2018_894_MOESM10_ESM.tif]
